# Vaccine preferences and their role for vaccine confidence and uptake: a meta-ethnography

**DOI:** 10.1080/16549716.2025.2588846

**Published:** 2026-02-13

**Authors:** Jeniffer Landicho, Thea Andrea Bravo, Jonas Wachinger, Catherine Silvestre, Kate Bärnighausen, Kerry Scott, Till Bärnighausen, Shannon A. McMahon, Mark Donald C. Reñosa

**Affiliations:** a Department of Epidemiology and Biostatistics, Research Institute for Tropical Medicine – Department of Health, Muntinlupa, Philippines; b Heidelberg Institute of Global Health, Ruprecht-Karls-Universität Heidelberg, Heidelberg, Germany; c School of Global Health, Faculty of Health, York University, Toronto, Canada; d Department of Global Health and Population, Harvard T.H. Chan School of Public Health, Harvard University, Boston, USA; e Africa Health Research Institute, Somkhele and Durban, South Africa; f Department of International Health, John Hopkins School of Public Health, Baltimore, MD, USA

**Keywords:** vaccines, vaccine preference, vaccination, vaccine confidence, vaccine uptake, policy

## Abstract

Vaccine confidence and uptake are influenced by individuals’ preferences regarding vaccine composition, quality, or administration pathways. However, literature synthesizing available qualitative insights into individuals’ vaccine preferences remains limited. We therefore conducted a meta-ethnographic systematic review of the qualitative literature on vaccine preferences to identify opportunities for enhancing vaccine confidence and uptake. We implemented a comprehensive search strategy and screened 5,528 studies across seven research databases published between 2001 and 2023. We identified and synthesized 97 qualitative articles to delineate factors influencing consumers’ vaccine preferences. Our findings revealed four primary domains shaping individuals’ vaccine preferences: Product, Place, Price, and Promotion. First, individuals’ preferences for vaccines often hinge on perceived quality and safety of the product itself, which can, for example, be associated with vaccine brand or origin, especially in the case of novel vaccines. Second, people prioritize convenience in terms of vaccination sites and delivery methods (wanting vaccinations offered at their doorstep or in local peripheral clinics); evidence regarding preferred groups to administer the vaccines was mixed. Third, the price of vaccines and the secondary costs associated with vaccination played a role in uptake considerations. Finally, both the sources of information (such as healthcare workers, community volunteers, and religious authorities) and the methods of promoting vaccine information (including face-to-face consultations during clinic visits and the distribution of leaflets or banners), emerged as crucial factors shaping decision-making processes. Overall findings highlight the importance of addressing multifaceted preferences to enhance vaccine confidence and uptake. By understanding individuals’ vaccine preferences, strategic recommendations can be developed to optimize vaccination programs and ensure acceptability and utilization.

## Paper Context

- **Main findings:** This review highlights key factors that influence vaccine preferences, such as the quality of the product, accessibility of vaccination sites, pricing considerations, and the effectiveness of promotional efforts.
- **Added knowledge:** We provide an in-depth analysis of how marketing principles, particularly the 4Ps (Product, Place, Price, Promotion) can shape the acceptance and utilization of vaccines among the public.
- **Global health impact for policy and action:** These valuable insights offer essential guidance for policymakers and healthcare providers to develop reliable interventions that can bolster vaccine confidence, increase uptake, and eventually improve global vaccination coverage.

## Background

Vaccination stands as a cornerstone of public health, having saved an estimated 154 million lives worldwide since 1974 by preventing infectious diseases such as hepatitis, diphtheria, tetanus, pertussis, measles, and polio [1–3]. Vaccination programs are instrumental in advancing global health goals, including the Millennium Development Goals 4 and 5 (reducing child mortality and improving maternal health) and the Sustainable Development Goal 3, (ensuring healthy lives and well-being for all [4–6]. More specifically, from 1990 to 2013, under-five mortality fell by 49%, aided in part by improved immunization coverage, including a 74% reduction in measles deaths from 2000 to 2013 [5]. During the same period, maternal mortality has also dropped by 45% [5,7,8].

Despite these remarkable gains, public trust in vaccines has been increasingly undermined: already prior to the COVID-19 pandemic, vaccine hesitancy and controversies posed significant threats to public health [9]. In 2019, the World Health Organization (WHO) identified vaccine hesitancy–defined as the reluctance or refusal to vaccinate despite vaccine availability–as one of the top ten global health problems [10]. The challenge of vaccine hesitancy is especially acute in low- and middle-income countries (LMICs), where fragile health systems and resource limitations compound the risks associated with vaccination coverage [11–13].

Efforts to strengthen vaccination programs and improve vaccine uptake require a nuanced understanding of the factors shaping individual decision-making. While vaccine hesitancy, confidence, and uptake are distinct constructs, they are closely related [14–17]. Vaccine confidence generally refers to trust in vaccines, the systems that deliver them, and the motivations of policymakers and individuals [17–19]. Low confidence may contribute to hesitancy, which in turn may lead to reduced uptake [20]. However, several studies have shown that vaccine hesitancy is additionally shaped by a range of factors beyond confidence, including past experiences with health services, perception of disease risks, and how information is communicated. Contextual influences such as historical, political, and socio-cultural, also play a role in how individuals perceive and engage with vaccines [21–24].

Vaccine uptake, meanwhile, can be influenced by practical access barriers, even when confidence is high [25]. Existing systematic reviews have elucidated various barriers affecting vaccination uptake [26–29], including individual preferences regarding vaccination design and delivery [27,30]. These studies span diverse vaccine contexts—such as childhood immunizations [26,27], global perspectives on routine vaccination [28], influenza vaccines [29], and HPV vaccination [30]—and highlight the role of individual experiences and service design in shaping decisions. However, these reviews tend to center on barriers or determinants of uptake rather than vaccine preferences per se, and are predominantly quantitative in nature. In contrast, preferences [31]—understood as people’s stated or inferred inclinations toward certain attributes—represent an emerging area of interest in the vaccination literature. Despite their increasing salience in public discourse, vaccine preferences remain undertheorized and underexplored within the academic literature.

In this article, we define vaccine preferences as individuals’ stated or inferred inclinations toward particular attributes of vaccines (e.g. brand, delivery method, dosing schedule, site of administration), shaped by both personal and contextual factors [32,33]. These preferences reflect affective, cognitive, and social processes, and are often embedded in cultural expectations, past experiences, and media messaging [34]. While many studies use discrete choice experiments or survey methods to assess the relative importance of specific vaccine attributes [30,33,35–38], preferences are not static; rather, they are dynamic, subjective experiences, and culturally mediated–evolving through complex interactions with societal norms, personal experiences, and environmental influences [39].

This nuanced perspective on preferences, rooted in disciplines such as social psychology and anthropology [34], emphasizes that preferences extend beyond a mere evaluation of product features [30,35,36,40,41]. Understanding individuals’ preferences necessitates a deeper examination of how individuals develop, negotiate and articulate their desires within broader cultural and social contexts. Such an approach would offer a comprehensive insight into how people constructs, mediate and express these preferences in their everyday lives.

In the context of vaccination, preferences are influenced by geographical location, cultural background, religious beliefs, education, media messaging, and service delivery options among others [21]. For example, in many Muslim-majority countries, vaccine content and the influence of religious leaders significantly determined community decisions and attitudes toward vaccination [42]. During the COVID-19 pandemic, vaccine brand preference became a significant phenomenon in several countries–sometimes driven by perceived efficacy, nationalism, rumors, and misinformation [43–46]. Additionally, vaccine brand preferences were influenced by vaccine presentations in diverse social media content [46,47]. When these preferences were unmet or ignored, public confidence and trust in the vaccination program was often undermined–even when vaccines were accessible and free [45]. In this way, vaccine preferences may serve as signals or expressions of deeper sentiments–such as trust, fear, or exclusion–that intersect with vaccine confidence and shape hesitancy [24]. Conversely, when available vaccine options align with people’s preferences, this may reinforce confidence and increase uptake. Understanding preferences requires more than mapping feature rankings; it calls for contextualized, qualitative insights into how individuals interpret and express what matters to them in relation to vaccination.

In this article, we present findings from a meta-ethnographic systematic review of available qualitative literature that examines how individuals experience, negotiate, and articulate preferences related to vaccines and vaccination. Rather than assuming preferences directly cause or prevent hesitancy, we explore how preferences interact with broader social and institutional factors to shape vaccine intention and, in some cases, uptake. Recognizing and addressing individuals’ vaccine preferences is crucial for informing policies and improving immunization programs, and ensuring vaccination efforts are responsive, trusted, and contextually grounded.

## Methods

We conducted a meta-ethnographic systematic review informed by Noblit and Hare [48]. Meta-ethnography is useful for synthesizing qualitative research and creating frameworks to analyze and understand the results from various studies [49,50]. Unlike other qualitative synthesis approaches, which may focus more on aggregating themes or describing patterns, meta-ethnography enables the interpretive translation of concepts across primary studies. This results in new understandings or frameworks that are more explanatory and conceptually rich–an approach especially appropriate for complex, value-laden topics such as vaccine preferences, where context and meaning shape individual decisions [51,52].

Our review followed a seven-step framework [48]: 1. Getting started; 2. Deciding what is relevant to the initial interest; 3. Reading the studies; 4. Determining how the studies are related; 5. Translating studies into one another; 6. Synthesizing translation; and 7. Expressing the synthesis. In the following sub-section, we outline how we performed each of these steps within our study.

### Getting started

JL and MDCR began with a brainstorming session to ‘identify the intellectual interest’ within the area of vaccine preferences [48]. Preliminary research questions were sent to the broader team for evaluation of relevance and acceptability. Following a series of discussions and iterative refinement, we finalized the overall question, which was to examine vaccine preferences from the perspective of the public.

### Deciding what is relevant to the initial interest

After the primary question was finalized, JL, TAB and MDCR convened to outline core concepts and main keywords to be used to develop the search string strategy. To create the initial search strings, we combined Medical Subject Headings (MeSH) and Non-MeSH terms such as *‘Vaccine Preference’, ‘Vaccine Attributes’, ‘Vaccine Acceptability’, ‘Qualitative studies’* and *‘Qualitative Research’*. The keywords were combined using Boolean operators like ‘AND’, ‘OR’, and ‘NOT’. We tested the validity of our initial search string strategy by conducting a pilot search on PUBMED and ScienceDirect to refine our search strategy and identify any remaining issues. We evaluated the number of search result and assessed their relevance to the study. Afterward, we extracted the MESH terms from the identified relevant studies. We chose to adopt the broader subject heading so that all terms nested under it would be included in the search, resulting in a comprehensive coverage of relevant literature, which is detailed in Supplementary Table S1.

After the search string strategy was finalized, JL executed the final search strings across six primary research databases including PubMed, ScienceDirect, ProQuest, EBSCOhost, JSTOR, and Scopus. To further enhance the comprehensiveness of our search, a secondary search was conducted using Google Scholar to capture grey literature and additional relevant studies. TAB then independently re-ran the final search strings across five research databases to validate the search results, confirming consistency and accuracy. The results from each search string of JL were exported and tabulated per research database using Endnote (version 8.2) and Microsoft Excel (version 2019), facilitating efficient documentation and removal of duplicate references.

After JL removed duplicates, the MS Excel sheet was shared with the other members of the research team. JL, TAB and CS independently screened the titles and abstracts of papers retrieved from the seven research databases (both primary and secondary databases) based on pre-defined eligibility criteria (see Table 1). Further, we performed a reference listing of all included articles to identify additional publications that may have been missed in our initial search combinations.

**Table 1.** Inclusion and exclusion criteria.

Criterion	Inclusion	Exclusion
Literature focus	(1) Original research (2) Research involved qualitative methods (3) Findings included qualitative insights on vaccine preferences	(1) Quantitative studies (2) Systematic reviews (3) Studies without ethical approval (4) Review articles, commentaries, conference or seminar papers and/or personal viewpoints (5) Articles that are abstract only and/or no retrievable full text (6) Books/Theses/Dissertation (7) Vaccine preference studies on veterinary vaccines
Time period Language	January 2001* – August 2023 English	

*Reflects the timepoint when several new vaccines (varicella and other conjugated vaccines) were introduced for the general public, sparking debates about vaccine preferences.

### Reading the studies

To familiarize ourselves with the content of the studies, JL and TAB read and re-read all included studies. Using printed copies of the studies, we manually highlighted and annotated key information using markers and highlighters. This collaborative, hands-on approach enabled close engagement with the data and supported the identification of relevant concepts and contextual elements. An evidence table was developed to systematically extract and record pertinent contextual data from each study.

To enhance process rigor, MDCR reviewed four studies and compared their observations with those of JL and TAB. This step helped to validate the coding and data extraction approach before the two lead authors proceeded to apply the same process across all remaining included studies.

JL and TAB assessed the quality of all included studies using the Consolidated Criteria for Reporting Qualitative Studies (COREQ). This appraisal focused on evaluating the transparency and rigor of study reporting but did not inform study inclusion or exclusion. Instead, it was used to support a more nuanced interpretation of the findings during synthesis.

### Determining how studies are related

We performed manual coding to highlight and compare relevant statements (i.e. all key metaphors, ideas, and/or concepts and their relations) across articles. These statements were tabulated in the excel sheets with reflexive notes or memos to help us bridge some nuances or confusion with the data and support us in the finalization of the codebook.

To promote reflexivity and ensure that the coding accurately reflected the core content and interpretations of the included studies, we employed several strategies. First, we engaged in regular discussions as a research team to reflect on our positionalities, disciplinary backgrounds, and potential biases that could influence the interpretation of the data. These reflexive discussions were particularly important given our interdisciplinary team, which included public health researchers and social scientists. Second, to assess the trustworthiness of our thematic coding and promote intersubjectivity, four articles were randomly selected and independently coded by JL and MDCR. The results were then compared with the codes and themes generated via a meeting. Discrepancies were discussed, and convergence across interpretations was used to refine the codebook iteratively. This dual process of reflexive dialogue and collaborative coding contributed to a more nuanced and transparent analysis. This process supported us in the finalization of the codebook.

### Translating the studies into one another

We employed a layered approach to generate new and higher-order interpretations that drew on both first- and second-level analysis. At the first level, JL, TAB, and MDCR extracted illustrative participant quotes from the included studies, which served as our primary data. These quotes were systematically coded, and similar codes were clustered to identify patterns and develop emerging concepts across studies. To complement this, JL and MDCR also engaged in second-level analysis by considering the themes, categories, and interpretations offered by the original study authors. This allowed us to compare not only what participants said, but also how study authors understood and contextualized those views. This dual-level approach enabled us to surface both the direct expressions of vaccine preferences and the broader interpretive insights embedded in the primary literature.

The coded data, accompanying analytical memos, and emerging themes were then compared across all included studies to identify patterns and variations in how vaccine preferences were described. This process allowed us to gain an overview of how key elements of vaccine preferences emerged and interacted across diverse settings and populations. Each theme was carefully reviewed to ensure conceptual clarity, distinctiveness, and coherence. To further strengthen transparency and rigor, we held collaborative team discussions throughout the analysis process. These discussions helped guide interpretative decisions, drawing upon existing literature and our own contextual knowledge, and informed our decision to map the inductively developed insights in the later stages of analysis.

### Synthesizing translations and expressing the synthesis

Our analysis followed an iterative and layered process. We began with an inductive thematic analysis to identify patterns and themes emerging directly from the data, allowing participant perspectives to shape the initial structure of our findings. As these core themes became well-established, we observed that many of them related to the characteristics of the vaccine itself, the information available about it, associated costs, and vaccination access.

To further deepen our interpretation and enhance analytical coherence with existing literature, we then organized the emerging themes into analytical categories using Borden and McCarthy’s ‘4Ps of Marketing Mix’ framework, as a secondary, deductive lens [53]. This framework facilitates the identification of crucial factors that contribute to the attractiveness of a product or service (in our case, vaccines and vaccination programs) to clients, focusing on the 4Ps–Product, Place, Price, and Promotion. We mapped the inductively derived themes onto the 4Ps domains, enabling us to organize the findings in a way that preserved the originality of participants’ insights while providing a structured, theory-informed basis for understanding the broader drivers of vaccine preferences [54].

## Results

### Characteristics and data sources of included studies

**Figure 1.**
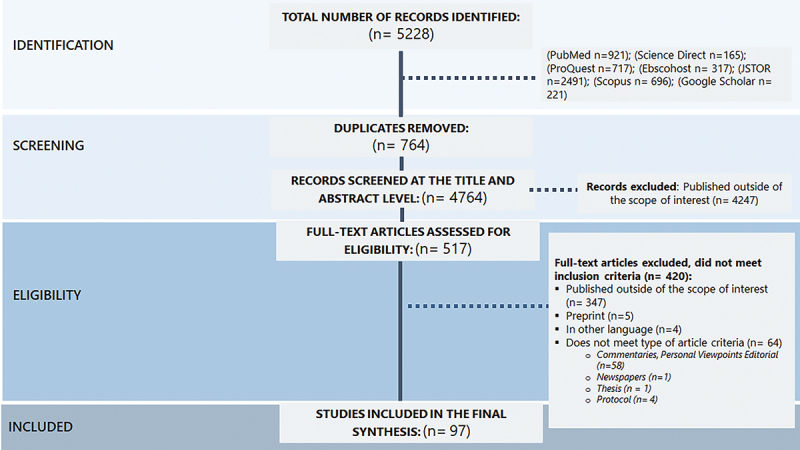
PRISMA diagram of articles identified and selected.

We performed a comprehensive review of literature published between January 2021 and August 2023, identifying a total of 5,228 studies. After removing 764 duplicates, we screened titles and abstracts of 4,764 and reviewed the full text of 517 studies. After screening and assessing study eligibility, our final sample consisted of 97 qualitative studies as shown in Figure 1.

Of the included studies, 65% were conducted in high-income countries (HICs), including the USA (*n* = 19), UK (*n* = 13), Canada (*n* = 10), the Netherlands (*n* = 4), New Zealand (*n* = 2), Switzerland (*n* = 2), Australia (*n* = 7), Spain (*n* = 1), Poland (*n* = 1), and Sweden (*n* = 1). Studies conducted in upper-middle-income countries accounted for 9% of the total while those in low- and middle- income countries (LMICs) represented 14%. Low-income Countries (LICs) were represented in 4% of the included articles, specifically Uganda (*n* = 3) and Sierra Leone (*n* = 1). Additionally, six (6%) of the included studies used a multi-country setting, such as HICs and LMICs or more specific pairings such as LMICs and LICs, or HICs and upper middle-income countries (UMICs). These countries were categorized following the World Bank Classification, which categorizes countries into four income groups based on the previous year’s Gross National Income per capita [55].

Forty-six percent of the included studies employed a descriptive qualitative study (*n* = 45) followed by an exploratory qualitative study (*n* = 28; 29%). Remaining studies were either cross-sectional qualitative, phenomenology, grounded theory, or other types of qualitative study design. To identify and recruit the study participants, 73% of the included studies used purposive sampling (*n* = 71), Respondent groups included parents, adolescents, the elderly, healthcare providers, and policymakers. Additionally, three studies specifically included indigenous peoples, and two studies involved religious leaders. Data collection methods across included studies were interviews (*n* = 52) and focus-group discussions (*n* = 24), with many studies (*n* = 21) using a combination of both methods and other techniques such as observation and qualitative surveys. Thematic analysis (*n* = 57) was the most common analytical approach, followed by content (*n* = 12) and framework analysis (*n* = 12). For a detailed description of the included studies, please refer to *Supplementary Table S2*.

### Quality of included studies

Across COREQ domains related to research team composition and reflexivity, 26 out of 97 studies reported the researcher’s credentials, and 31 studies indicated the researchers’ occupation at the time of research implementation. Additionally, 38 studies specified the researcher’s experience and training in conducting qualitative research (*see Supplementary Table S3*).

Many of the included studies (*n* = 64) described their methodological orientations and theoretical frameworks and provided details on participant involvement and selection processes. However, only 29 studies reported reasons why approached individuals declined to participate or dropped out prematurely, and nine studies did not specify how participants were approached. Data saturation was discussed in 46 studies. In terms of data analysis, 69 studies reported the number of coders, with 51 of these articles providing descriptions of the coding tree. Most studies (*n* = 63) used qualitative data analysis software such as NVivo, Atlas.ti, and MAXQDA.

### Key findings

**Figure 2.**
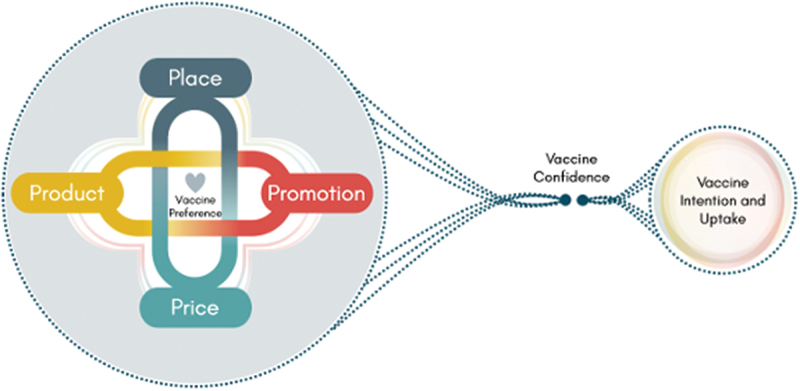
Vaccine preferences among the general public.

We identified various themes via the synthesis of included studies, which we organized based on the core concepts of the 4Ps: Product, Place, Price, and Promotion [53]. While many studies primarily focused on discussions surrounding HPV and COVID-19 vaccines, our analysis highlights the broader complexities of vaccine confidence and uptake across different vaccines, including routine childhood vaccines, HPV, COVID-19 vaccines, and other adult vaccines. Figure 2 illustrates these complexities, emphasizing how preferences and decision-making are influenced by a range of factors.

A large proportion of identified preferences centered on the product (i.e. the vaccine) itself, encompassing considerations of quality, safety, composition, and administration details e.g. dosage). Additionally, the location or place of vaccination (e.g. government or private health facilities), including timing and availability, influenced respondents’ vaccine decision-making. Fewer articles highlighted strong preferences related to vaccination price, with considerations commonly focusing on the cost of new vaccines not (yet) covered by governments or insurers, and indirect expenses such as transportation costs or lost income from missed work. In comparison, the promotion of vaccines featured more prominently in the identified articles, particularly in terms of vaccine information considerations and the preferred sources for this information.

**Table:** 

4Ps	Vaccine Preferences	Factors influencing preference	Article Reference/Country
Product	Vaccine Quality	Extensive product research and qualityassurance testing	Australia [74]
			Canada [62]
			Canada [75]
			HongKong SAR [57]
			Hongkong SAR [61]
			Hongkong SAR [69]
			India [72]
			Malaysia [56]
			New Zealand [58]
			The Netherlands [59]
			Uganda [63]
			United Kingdom [60]
			United Kingdom [73]
			United States [64]
			United States [65]
			United States [66]
			United States [67]
			United States [68]
			United States [70]
			United States [71]
		Demonstrate high efficacy with favorable safety and tolerability profile	Australia [74]
			Australia [91]
			Australia [102]
			Canada [62]
			Canada [77]
			Canada [83]
			Canada [84]
			Canada [88]
			China [101]
			Haiti [85]
			Hongkong SAR [61]
			HongKong SAR [69]
			India [97]
			India [100]
			Multi-country [121]
			Multi-country [95]
			Nigeria [82]
			Tanzania [96]
			The Netherlands [78]
			The Netherlands [92]
			Uganda [90]
			Uganda [99]
			United Kingdom [79]
			United Kingdom [80]
			United Kingdom [81]
			United Kingdom [87]
			United Kingdom [98]
			United States [67]
			United States [76]
			United States [89]
			United States [93]
			United States [94]
			United States [71]
			United States [103]
			Venezuela [86]
		Clinically effective with extended duration of protection	Australia [91]
			Canada [83]
			Canada [84]
			China [104]
			Haiti [85]
			HongKong SAR [57]
			Nigeria [82]
			Sierra Leone [110]
			Switzerland [107]
			Switzerland [109]
			The Netherlands [59]
			The Netherlands [92]
			The Netherlands [105]
			Uganda [90]
			United Kingdom [79]
			United Kingdom [98]
			United States [65]
			United States [76]
			United States [94]
			United States [71]
			United States [108]
			Vietnam [106]
	Production, Packaging, Composition, and Administration	With Careful consideration of ethical standards and cultural values in the design and manufacturing	HongKong SAR [113]
			Indonesia [114]
			Malaysia [56]
			Multi-country [115]
			New Zealand [58]
			Sweden [111]
			The Netherlands [92]
			United Kingdom [79]
			United Kingdom [112]
		Desire to minimize the frequency and volume of vaccine administration	Australia [91]
			Canada [116]
			HongKong SAR [57]
			Malaysia [56]
			Tanzania [96]
			United Kingdom [117]
			United Kingdom [118]
			United States [76]
Place	Adequate supply of vaccines andaccessibility of service delivery point	With adequate supply of vaccines	Mexico [119]
			Panama [120]
			United Kingdom [73]
		Accessible service delivery point	Australia [91]
			Canada [75]
			Canada [124]
			Multi-country [121]
			Tanzania [123]
			United States [65]
			Zambia [122]
	Assurance of privacyand preference for well-trained healthcare providers	Private	United States [125]
		Well trained health care provider	Canada [116]
			Mexico [119]
			United Kingdom [117]
			United States [108]
			United States [125]
Price	Vaccination cost Or Preference for affordable vaccination options and trusted vaccine brands	Affordable Vaccination cost (High prices i.e not free or affordable price, covered by insurance)	Canada [62]
			Canada [116]
			Côte d’Ivoire [129]
			HongKong SAR [57]
			Hongkong SAR [61]
			India [72]
			India [128]
			India [130]
			Malaysia [56]
			Multi-country [121]
			Multi-country [127]
			Nigeria [82]
			The Netherlands [59]
			United States [65]
			United States [66]
			United States [67]
			United States [126]
		Preferences to trusted vaccine brands	Australia [74]
			Australia [132]
			China [104]
			Côte d’Ivoire [129]
			Hongkong SAR [61]
			Multi-country [127]
			Poland [131]
			Singapore [134]
			United Kingdom [117]
			United States [135]
			Vietnam [133]
	Preference for Cost-effective vaccine that reduce financial and healthcare burden	Cost-effectiveness of the vaccines	Canada [77]
			India [100]
			India [128]
			India [136]
			Multi-country [121]
			United States [67]
	Reduction of secondary costassociated with vaccination	Secondary costs associated with vaccination	Australia [138]
			India [130]
			United States [137]
			Zambia [122]
Promotion	Preferred content of Vaccine information, sources and channels	Comprehensive informationon the value of vaccines	Canada [116]
			HongKong SAR [69]
			India [128]
			Malaysia [56]
			Mexico [119]
			Panama [120]
			Spain [141]
			The Netherlands [59]
			United Kingdom [70]
			United Kingdom [73]
			United Kingdom [139]
			United Kingdom [143]
			United States [67]
			United States [70]
			United States [94]
			United States [140]
			United States [142]
			Vietnam [106]
			Zambia [122]
		Trusted messengersin conveying vaccine information	Australia [102]
			Australia [144]
			Australia [145]
			Canada [83]
			Canada [84]
			Canada [88]
			Canada [147]
			Haiti [85]
			Hongkong SAR [61]
			HongKong SAR [69]
			HongKong SAR [113]
			India [128]
			Indonesia [114]
			Malaysia [56]
			Multi-country [121]
			Multi-country [146]
			Panama [120]
			United Kingdom [98]
			United States [67]
			United States [140]
			United States [142]
			Vietnam [106]
			Zambia [122]
		Preferredmode of delivery and channel of information	Canada [75]
			Canada [148]
			The Netherlands [59]
			United States [89]
			United Kingdom [117]
			United States [135]
			United States [140]
	Freedom of choice versus mandatory vaccination	Respect for autonomy of individual and freedom of choice	Austria [150]
			Canada [62]
			HongKong SAR [113]
			Multi-country [127]
			New Zealand [149]
			The Netherlands [78]
			Poland [131]
			United Kingdom [87]
			United States [64]
		Preference for Mandatory vaccination	United States [67]

## Product

### Vaccine quality

#### Extensive product research and quality assurance testing

In most studies (91 out of 97), participants highlighted their preference for a vaccine that was of high quality (commonly defined in terms of vaccine efficacy and effectiveness in preventing disease) and safe (commonly defined as the absence of or minimal side effects).

Across studies, participants emphasized the critical role of safety in evaluating the vaccine quality, especially for new vaccines. For instance, in a study on parents’ refusal of childhood vaccination in Malaysia, one participant emphasized a need for vaccine safety ‘to be guaranteed through adequate testing and research, until doctors can give the assurance that it is 100% safe’ [56]. Studies conducted in Hong Kong, New Zealand, and the Netherlands also highlighted concerns among parents and young adults regarding the insufficient investigation of new vaccines before their introduction to the global market [57–59]. Similar suspicions and concerns arose during emergencies when vaccines were perceived to be manufactured and deployed prematurely without adequate scientific scrutiny, as observed with the AH1N1 vaccine [57]. In a UK study exploring COVID-19 and COVID-19 vaccination beliefs, a participant voiced concerns regarding the rapid development of vaccines: ‘They’ve made it so quick we don’t know the side effects it’s going to have in the future’ [60]. The uncertainties surrounding new vaccines often led participants to prefer waiting and assessing the vaccine’s effects on others first [70–75,127]. Studies conducted in Hong Kong SAR, Canada, Uganda, and the U.S. indicated a prevailing preference to wait until long-term studies were available [61–67], which could identify limitations and possible side effects before broader roll-out [59,60,65,68,69]. Participants expressed concerns about the need for more studies on vaccines to address potential risks, as illustrated by a participant’s comment: ‘Do more studies on the vaccine. Sometimes they say the vaccine is good, and then a few years after that you hear someone died because of it or it is making things worse’ [67].

#### Demonstrate high efficacy with favorable safety and tolerability profile

Participants across studies consistently preferred vaccines with few to no side effects and high efficacy [61,62,67,69,71,76–87,102,121]. Many individuals set high standards for vaccines, voicing a preference for 100% efficacy, as evidenced in a Canadian study among Indigenous People [62]. Moreover, several studies underscored participants’ preference for vaccines with demonstrated long-term vaccination efficacy [76] and effectiveness in preventing vaccine-preventable disease [69,74,77–80,121]. If the vaccine is effective in preventing infections, some participants showed a willingness to accept short-term side effects (e.g. fever, injection site pain and scarring, stomach flu, dizziness, and fatigue) [61,67,69,76,82–89,103], highlighting complex trade-offs between immediate discomfort and long-term benefits. On the other hand, individuals who had either experienced the diseases firsthand or were more susceptible to diseases expressed willingness to accept vaccines with lower effectiveness [84,90–96], suggesting that their direct experience may make them open to any level of protection. However, concerns about severe side effects–such as death, deformities, disabilities, and vaccine effects on unborn children–were significant barriers to vaccine uptake [63,67,97–101].

#### Clinically effective with extended duration of protection

Participants across settings consistently emphasized that a high-quality vaccine is one that effectively prevents disease and offers protection [57,59,65,68,76,79,82–85,90–92,94,98,104–108]. For instance, in Vietnam, a parent of a fully vaccinated girl stressed the importance of vaccines for rural residents who cannot afford healthcare [106], while a Swiss participant described vaccination as essential, saying ‘vaccinating is life’ [107,109]. Parents, especially those with children in school, expressed that vaccinating their children alleviates their anxieties, as vaccines help mitigate the risks of preventable diseases. Beyond individual-level factors, some participants also recognized the broader societal benefits of vaccination, particularly its role in disease eradication [71,96,98,99,110].

### Production, packaging, composition and administration good manufacturing product

#### Designed and manufactured with consideration of ethical standards and cultural values

Participants’ perspectives on vaccine content and manufacturing methods including parenteral containers and dosage forms differed considerably across studies and countries [103,115,130,151]. In the Netherlands, Sweden, and the UK, participants expressed fears about the potential use of harmful substances such as chemicals, metals, microchips, or viruses in vaccine production [92,111,112]. Participants from Malaysia, New Zealand, and Hong Kong voiced concerns about injecting ‘foreign’ substances into their bodies, perceiving potential harm [56,58,113]. Other participants questioned the cleanliness of the vaccine production process based on their religious beliefs, raising worries about the use of animals or aborted fetuses in an exploitative manner [56]. Muslim participants in clinical trials in Malaysia and the UK strongly preferred ‘halal vaccines’ that guaranteed the absence of any pig-derived fragments [56,79]. Similarly, participants in Indonesia sought approval from *ulamas* (Muslim scholars recognized for their expertise in Islamic law and theology) to ensure the acceptability and purity of the vaccine products [114].

#### Desire to minimize the frequency and volume of vaccine administration

Several studies highlighted preferences related to vaccine dosage, intervals, and administration schedules [103,130]. In a Tanzanian study on the acceptability of dosage reduction for HPV vaccines, nearly all girls preferred receiving the vaccine in a single dose [96]. Similarly, a Canadian study on attitudes toward a hypothetical HIV vaccine revealed that most participants preferred a single injection without booster follow-ups [116]. In Hong Kong, a mother mentioned her husband’s refusal to allow their daughter to receive additional vaccinations due to concerns about excessive injections [57]. Similarly, parents in the UK voiced concerns about children receiving multiple injections within a short period [117].

In addition to a preference for receiving a single-dose vaccine, preferences also emerged regarding combining multiple vaccines into a single injection, while participants also acknowledged potential challenges. In Australia, young women desired vaccines that could protect against multiple diseases but recognized the complexity of producing combined vaccines [91]. This assumption was mirrored in an American study among HIV high-risk groups where participants acknowledged that, while theoretically preferred, a single-dose vaccine protecting from all HIV subtypes might not be realistic or adequate as it might make developing the vaccine even more challenging [76]. In the UK, parents also expressed concerns about the limitations of the influenza vaccine, recognizing that it only protects against the prevailing strain in a given year and not against all potential strains [118]. In contrast to this, a Malaysian parent expressed a preference to avoid combination vaccines [56].

## Place

### Adequate supply of vaccines and accessibility of service delivery point

A study conducted among ethnic minority and White British groups revealed that they preferred to receive vaccines in community health care settings like hospitals, and other places that they deemed safe [73]. However, in a study on maternal vaccination in Mexico and Panama, participants mentioned occasions when public hospitals experienced vaccine shortages, prompting a desire to access both government and private health facilities [119,120]: ‘they (public hospitals) ran out of vaccines. They tell you to come back later or try other hospitals -or you can go to a private one’ [119]. Participants across several studies in the U.S., Greece, the UK and Canada [65,91,121,124] emphasized the importance of conveniently accessible vaccination locations: ‘You shouldn’t have to drive any longer than 20 minutes to access a vaccine’ [91].

In Zambia and Canada, participants proposed implementing door-to-door vaccination programs to target individuals who are sick or have disabilities [75,122]. Additionally, parents of teenagers eligible for HPV vaccination expressed a preference for school-based immunization programs due to their straightforward logistics [123].

### Assurance for privacy and preference for well-trained healthcare providers

Some participants in the U.S. expressed privacy concerns [125] and reservations about the level of training received by healthcare workers (HCWs) in public vaccination settings [108]. Similarly, parents in the U.S. preferred not to have their children vaccinated at community pharmacies, citing concerns about the clinical expertise of pharmacists [108]. Participants in Canada, Mexico and the U.S. preferred receiving vaccines at the clinic run by their healthcare providers [116,119,125]. In the UK, members of Polish and Romanian communities raised concerns about the qualification of nurses administering the vaccines, as vaccines in Poland are exclusively administered by medical doctors [117].

## Price

### Affordable vaccination option and trusted vaccine brands

Participants expressed hesitancies to avail of vaccines due to high prices, particularly if their income was limited [57,59,127]. In both Hong Kong and the U.S., participants with multiple children preferred to know the required dosage regimen beforehand due to concerns about potential expense [57,66,126]. Several studies concluded that vaccines should generally be given for free [62,72,82,121], or at least at an affordable price [57,65,82,128,129], irrespective of citizenship or life situation [116]. Purchasing vaccines could be quite costly, causing some people to decline, especially if they believe they are already immune to these diseases [57,61].

Respondents in the U.S. studies indicated that vaccine coverage through health insurance would serve as a significant driver for uptake [66,67,126]. Furthermore, participants in Malaysia preferred life-long governmental compensation to those who suffered from severe vaccination side effects to alleviate fears of catastrophic healthcare expenditure following vaccination [56]. Brand preferences also influenced the vaccination costs. Participants in various studies expressed preferences for specific vaccine brands or types not commonly available in public health facilities due to concerns about the quality of the vaccines [74,117,127,129,131], i.e. people have to pay more because they want a specific vaccine which is not covered by the government [57]. Brand-conscious people wanted to know which vaccine brands were more suitable [61,104,135] and this was particularly evident during the COVID-19 pandemic, where preferences were shaped by perceptions of certain brands as safer, more effective, or more trustworthy than others [132–135].

### Preference for a cost-effective vaccine that reduces financial and healthcare burden

Participants in studies across Canada, Australia, the U.S., and India expressed a willingness to spend money on vaccines that would protect them from diseases [67,77,121,128]. They preferred the upfront cost of vaccination over potentially higher expenses associated with disease management and treatment at a later time [67,100]. This perspective on the cost-effectiveness of vaccination was echoed by a community member in India, where antenatal influenza vaccination was seen to prevent disease and reduce healthcare costs: ‘If she is vaccinated prior and her immunity increases, then she won’t suffer from these diseases. That means it will not increase the financial burden for healthcare, and patient’s expenses are saved’ [136].

### Reduction of secondary costs associated with vaccination

Participants across studies in India, Zambia, U.S., and Australia also voiced preferences regarding vaccination schedules that minimize indirect costs [122,130,137,138]. In Zambia, a study was conducted to investigate the impact of vaccines in informal settlements. The study found that while most participants preferred following conventional vaccination schedules, some individuals, particularly men, expressed a preference for receiving vaccines on Saturdays and Sundays, when they were not required to work [122].

## Promotion

### Preferred content of vaccine information, sources and channels

Vaccine information influences an individual’s decisions regarding vaccine uptake and acceptance, as highlighted in several studies [85,87,92,120,125,128,131,141–144,152]. To make informed decisions, participants in the U.S., Mexico and Panama expressed a desire for comprehensive information about the value of vaccination [70,94,119,120]. They preferred to know details such as the type of vaccine, how it works, diseases it prevents, dosage regimen, side effects, benefits, and risks [56,59,69,106,116,122,139,140]. Participants emphasized the importance of receiving accurate and transparent information, with one British participant suggesting: ‘ … I think it would make it easier if it was true information, stating that it was for this strain of flu and this is what’s happened to people that have had it and people that haven’t had it’ [139]. This sentiment was echoed by a Haitian participant who regretted consenting to her child receiving the HPV vaccine due to feeling uninformed at the time [67].

Trusted messengers were identified as crucial in conveying vaccine information effectively [83,146]. Yet, who was considered a trusted source varied considerably even within populations. Studies in Canada, the UK, and Vietnam found that participants sought information from healthcare providers, while others preferred information from newspapers, social media, and mainstream media [73,88,98,106]. Participants valued HCWs who patiently explained vaccine purposes while emphasizing both the benefits and potential side effects, such as symptoms and complications of the underlying disease [56,61,140,145]. Participants across several studies expressed reliance on medical doctors for health decisions due to trust and confidence in their expertise [67,69,83–85,88,102,113,120,121,142,144,146,147].

Additionally, community volunteers and religious authorities were also recognized as trusted sources of vaccine information [128]. In Zambia, community volunteers supported vaccination efforts by addressing community concerns, easing fears, and creating spaces where people could openly discuss their reservations [122]. Participants also emphasized the importance of involving church leaders in vaccine information dissemination, recognizing their trusted role within the community and their ability to reach the congregation effectively [114,122].

Meanwhile, in the Netherlands, participants preferred traditional methods of information delivery, such as face-to-face consultations and the distribution of vaccine leaflets [59]. Canadian HCWs identified multiple channels for advertising information, including school-based campaigns, large banners, smartphone and television messaging, and vaccine reminder systems [148]. Some studies also highlighted preferences for vaccine information in multiple languages and presented in accessible language without scientific jargon [59,75,89,117,135,140].

### Freedom of choice on mandatory and optional vaccines

Across the included studies, many participants regarded childhood vaccinations as mandatory, aligning with their country’s national immunization program [59,131]. However, when it came to optional vaccines–such as the HPV vaccine, which was not required for school entry–some participants expressed a preference for exercising their personal autonomy and making their own vaccination decisions [64]. In the U.S., African American women felt that the HPV vaccine should not be mandatory [67]. The freedom of choice was also echoed for maternal vaccination from a participant from the Netherlands [78] who nevertheless acknowledged that freedom of choice should not always prevail. While several studies found strong preference for autonomy in decision-making [64,67,78,87,150], with many arguing that mandatory vaccination would juxtapose with freedom of choice [113,127,149], there was no concensus with optional vaccines, indicating complexity and challenges associated with balancing individual autonomy and public health objectives.

## Discussion

Our article synthesizes qualitative studies examining general populations’ vaccine preferences. Our findings outline vaccine preferences across all four domains of the 4Ps framework: Product, Place, Price, and Promotion. Product-associated preferences relate to vaccine quality, safety, and specific brands or origins, particularly for new vaccines. Place-associated preferences underscore the importance of vaccination settings, such as a doctor’s clinic or community health center, and the perceived competence of the vaccine administrator. Price-related considerations, particularly affordability and cost-effectiveness, play a crucial role in shaping vaccine acceptance, especially for new vaccines, as they significantly influence the level of trust and willingness to adopt. Promotion preferences included specific side-effect information dissemination, one-on-one methods of communication, and the involvement of credible messengers (e.g. physicians).

Our findings align with earlier systematic reviews that have explored ethical considerations in vaccine development and vaccination process [153] and factors influencing acceptance [33,154]. In our meta-ethnography, the quality and safety of vaccines emerged as primary preferences for vaccination. However, individuals often seemed to set an exceptionally high bar for vaccine quality, efficacy, and safety without actually considering the broader public health benefits [155]. Specifically, participants often prioritized the perceived risks of potential side effects from vaccines over the dangers of remaining unvaccinated [58,156,157], particularly for novel vaccines. Consequently, when individuals receive a vaccine (and endure the side effects) but later contract the infection, they tend to perceive that the vaccine has failed, reinforcing their skepticism. This asymmetry in risk perception is critical for understanding vaccine hesitancy and poses an opportunity for public health messaging [14]. Although vaccinated individuals may still contract the infection, the critical point is that they are substantially less likely to develop severe disease or require hospitalization [158,159]–a distinction that is essential for the public to have a clear comprehension of and trust in vaccines [160,161].

Our findings also highlight that individuals who opted for vaccination often emphasize the direct benefits for themselves or their children, such as protection during pregnancy or against HPV [157]. In contrast, societal benefits–including protecting others, reducing community disease burden or contributing to herd immunity–were mentioned less frequently. Although a study expanded the view of vaccine safety to include minimizing side effects and supporting overall health [162], the prevailing emphasis remained on individual rather than the societal health gains. While demand-creation approaches are broadly necessary to close vaccination gaps, our findings suggest that promotional messages highlighting personal protection may resonate more strongly with individuals than appeals to societal benefits [157]. Nonetheless, balancing these perspectives remains critical, particularly for novel vaccines such as COVID-19, which were recommended for entire populations–even those at low personal risks–precisely because of their population-level value [163].

The public’s confidence in the scientific basis of vaccination may not extend to new or innovative vaccines, even if these are based on well-established technologies [155]. This may indicate a more general doubt or lack of trust in the advancement of healthcare, especially when it pertains to novel or unfamiliar products [141]. Notably, in our meta-ethnography, vaccine composition, particularly vaccine ingredients, was a major consideration for novel vaccines, as individuals sought assurances that vaccine would not conflict with their cultural or religious beliefs. This aligns with a study conducted in Islamic-majority countries [42], where individual concerns about safety and religious compatibility outweighed attention to broader societal considerations. This aspect highlights the importance of transparency in presenting clinical trial data and stages of vaccine development, particularly in regions with diverse cultural and religious contexts. Communicating the continuity and shared principles between established and new vaccines, along with the critical role vaccines play in averting serious disease, could potentially narrow this divide.

In terms of vaccine development and composition, our review also highlighted a general preference for single-type vaccines over combined ones. The idea of receiving multiple shots within a short period of time to deliver different vaccines, as well as the concept of booster shots for the same vaccine, were often unpopular. This preference presents a significant challenge for vaccine developers, as reducing the number of shots typically necessitates combining vaccines into a single vial, and booster shots often are essential for maintaining vaccine effectiveness. Importantly, the reasons underlying the preference for fewer shots varied: in some cases, concerns centered on safety or discomfort, financial constraints, or the indirect costs of repeated clinic visits. These variations did not clearly align with specific country settings but rather appeared across individual- and structural-levels. While disentangling these influences was beyond the scope of this review, we emphasize that preferences should be understood as embedded within broader determinants of health-seeking behavior. Further research is recommended to explore the underlying causes and identify potential strategies to navigate trade-offs. Additionally, these findings highlight the need to refine public messaging strategies that more effectively communicates the benefits and safety of combined vaccines and multiple-shots vaccines.

While many studies have primarily focused on discussions surrounding HPV and COVID-19 vaccines [41,60,61,64–68,82,83,85,91,96,111,116,123,126,140,156,159,163–165], our findings underscore the contested and context-specific nature of vaccination narratives [166]. Vaccine preferences are shaped by multiple, intersecting factors, including individual’s current condition, prior experiences with vaccination, government implementation strategies, and broader public discourses (e.g. vaccine-related controversies) [167]. Our review not only identifies specific preferences but also sheds light on how these preferences are formed. For many, decisions are rooted in perceptions of vaccine quality and accessibility; however, uncertainties around novel vaccines were magnified by inconsistent and often conflicting public health messaging, which in turn eroded confidence [166]. Traditional and social media played a role in this process, as people actively sought information on safety profiles and potential side effects–a finding also observed in other systematic reviews [27,33]. The COVID-19 pandemic intensified the fragility of vaccine confidence, as misinformation spread rapidly and undermined acceptance of new vaccines [157]. Taken together, these findings underscore the importance of rebuilding trust through clear, transparent, and consistent communication strategies that addresses both safety concerns and misinformation, thereby reinforcing the foundations of vaccination programs [168].

In addition to identifying specific vaccination preferences, our review also provides insights into how such preferences are shaped by contextual factors. In LMICs, accessibility and vaccine availability often emerged as paramount considerations, with individuals’ choices closely tied to whether vaccines could be obtained easily and reliably. By contrast, studies from HICs suggested that location of vaccination was rarely a primary concern, except in circumstances where privacy or convenience became salient. Consistent with findings from other studies [165,169], vaccine supply is a critical factor influencing vaccine uptake across settings. Fewer studies directly explored perceptions of vaccine price (and its associated secondary costs) as a determinant of uptake [170,171]. Since a majority of routine vaccines are typically included in government immunization programs, the uptake of vaccines excluded from these programs will, among other factors, depend on their price. Some studies indicate that efforts to overcome the barrier of vaccination accessibility due to cost are yielding beneficial results [82,91,121]. Therefore, the discussion of vaccine equity and supply chain sustainability warrants further evaluation.

Finally, our analysis highlights expressed vaccine preferences but does not always capture the underlying reasons that shape them. For example, the preference for single-dose over multi-dose vaccines may stem from very different concerns depending on context–ranging from accessibility, affordability to work-related opportunity costs or broader socio-cultural factors. Preferences, therefore, should not be interpreted as root causes of hesitancy or acceptance, but rather as proximal expressions of deeper structural and contextual dynamics. While our synthesis captures vaccine preferences across these domains, our findings suggest that these preferences are not necessarily direct underlying drivers of vaccine hesitancy or uptake or confidence, but rather as expressions of how individuals navigate broader structural and contextual factors. Understanding preferences offers valuable insight into how individuals articulate their priorities when navigating vaccination decisions, even if these do not align neatly with the fundamental drivers of uptake or confidence. Future research is needed to further disentangle these interactions and clarify the mechanisms linking preferences with actual vaccination behaviors.

### Review strengths and limitations

This meta-ethnographic review provides evidence regarding the general population’s vaccine preferences, drawing on comprehensive searches across several databases with extensive search strings and overseen by a biomedical librarian (JL). However, some limitations must be considered. First, the literature search was restricted to studies published up to June 2023. This cut-off reflected the fixed timeline of the project and resource constraints, rather than an intentional exclusion of more recent work. Consequently, newer evidence published after this date could not be captured. Second, while our iterative methodology during literature extraction enabled a systematic examination of vaccine preferences and their relationship with confidence and uptake, the transferability of our findings may be constrained by the large number of studies conducted in HICs, the underrepresentation of vulnerable or marginalized groups, and variation in qualitative approaches used to explore vaccine preferences. Finally, as many studies were conducted in higher-income contexts, interpreting our results across more diverse settings remain challenging given the strong influence of context on perceptions.

## Conclusion

This meta-ethnographic review underscores the need to adopt a holistic approach when considering vaccination preferences in relation to intentions and uptake. Our findings suggest that policymakers and vaccine program managers must address contextual nuances and engage in collaborative efforts with diverse agencies to meet evolving vaccine preferences. Notably, there are significant gaps in the literature, particularly concerning vaccine preferences in LMICs. We hope for our findings to stimulate discussions on effectively integrating the general public’s vaccine preferences into existing vaccination programs, bridging the gap between preferences and actionable interventions.

## Supplementary Material

Supplementary Table 3_COREQ.pdf

Supplementary Table 2_Characteristics of Included Studies_Clean Copy_26 Aug 2025.docx

Supplementary Table 1_Summary of Research Databases.pdf

## Data Availability

The data used for this review are available upon request by sending an email to the first or the corresponding author.

## References

[1] CDC. Ten great public health achievements — United States, 2001–2010. 2011. 619–22. 21597455

[2] WHO. Raising awareness of immunization. World Health Organization. Available from: https://www.who.int/westernpacific/activities/raising-awareness-of-immunization

[3] Shattock AJ, Johnson HC, Sim SY, et al. Contribution of vaccination to improved survival and health: modelling 50 years of the expanded programme on immunization. Lancet. 2024;403:2307–2316. doi: 10.1016/S0140-6736(24)00850-X 38705159 PMC11140691

[4] WHO. Millennium development goals (MDGs). World Health Organization; 2018. Available from: https://www.who.int/news-room/fact-sheets/detail/millennium-development-goals-(mdgs)

[5] World Health Organization. Organisation mondiale de la S. Millennium Development Goals (MDGs). World Health Organization; 2018.

[6] Alliance GTV. Millennium development goals: gavi contributed to the eight millennium development goals 2020. updated 2020 Feb 18 [cited 2025 Jun 2]. Available from: https://www.gavi.org/our-alliance/global-health-development/millennium-development-goals#:~:text=MDG%205:%20improve%20maternal%20health,low%2Dincome%20countries%20were%20vaccinated

[7] Nations U. The millennium development goals report 2015. New York: United Nations; 2015.

[8] Maternal Immunization. Protected together. International Vaccine Access Center. 2020 [cited 2025 Jun 2]. Available from: https://immunizationevidence.org/maternal-immunization-protected-together/?utm_source=chatgpt.com

[9] de Figueiredo A, Simas C, Karafillakis E, et al. Mapping global trends in vaccine confidence and investigating barriers to vaccine uptake: a large-scale retrospective temporal modelling study. Lancet. 2020;396:898–908. doi: 10.1016/S0140-6736(20)31558-0 Epub 2020/09/14. PubMed PMID: 32919524; PubMed Central PMCID: PMC7607345. 32919524 PMC7607345

[10] WHO. Ten threats to global health in 2019. World Health Organization. Available from: https://www.who.int/news-room/spotlight/ten-threats-to-global-health-in-2019

[11] Hajizadeh M. Socioeconomic inequalities in child vaccination in low/middle-income countries: what accounts for the differences? J Epidemiol Community Health. 2018;72:719–725. doi: 10.1136/jech-2017-210296 Epub 2018/03/28. PubMed PMID: 29581228. 29581228

[12] Kaplan AD, Dominis S, Palen JG, et al. Human resource governance: what does governance mean for the health workforce in low- and middle-income countries? Hum Resour Health. 2013;11:6. doi: 10.1186/1478-4491-11-6 Epub 2013/02/19. PubMed PMID: 23414237; PubMed Central PMCID: PMC3584723. 23414237 PMC3584723

[13] Cobos Munoz D, Monzon Llamas L, Bosch-Capblanch X. Exposing concerns about vaccination in low- and middle-income countries: a systematic review. Int J Public Health. 2015;60:767–780. doi: 10.1007/s00038-015-0715-6 Epub 2015/08/25. PubMed PMID: 26298444. 26298444

[14] Badur S, Ota M, Ozturk S, et al. Vaccine confidence: the keys to restoring trust. Hum Vaccin Immunother. 2020;16:1007–1017. doi: 10.1080/21645515.2020.1740559 Epub 20200416. PubMed PMID: 32298198; PubMed Central PMCID: PMC7227637. 32298198 PMC7227637

[15] Roberts CH, Brindle H, Rogers NT, et al. Vaccine confidence and hesitancy at the start of COVID-19 vaccine deployment in the UK: an embedded mixed-methods study. Front Public Health. 2021;9:745630. doi: 10.3389/fpubh.2021.745630 Epub 20211111. PubMed PMID: 34858927; PubMed Central PMCID: PMC8632016. 34858927 PMC8632016

[16] Brackstone K, Marzo RR, Bahari R, et al. COVID-19 vaccine hesitancy and confidence in the Philippines and Malaysia: a cross-sectional study of sociodemographic factors and digital health literacy. PLoS Glob Public Health. 2022;2:e0000742. doi: 10.1371/journal.pgph.0000742 Epub 20221019. PubMed PMID: 36962550; PubMed Central PMCID: PMC10021455. 36962550 PMC10021455

[17] Williams CT, Saini B, Zaidi STR, et al. Determinants of high vaccine confidence and uptake among the Australian public: insights from a cross-sectional study. Front Public Health. 2025;13:1513892. doi: 10.3389/fpubh.2025.1513892 Epub 20250530. PubMed PMID: 40520270; PubMed Central PMCID: PMC12162927. 40520270 PMC12162927

[18] Quinn SC, Jamison AM, An J, et al. Measuring vaccine hesitancy, confidence, trust and flu vaccine uptake: results of a national survey of White and African American adults. Vaccine. 2019;37:1168–1173. doi: 10.1016/j.vaccine.2019.01.033 Epub 20190129. PubMed PMID: 30709722. 30709722

[19] Arsenault C, Ravishankar S, Lewis T, et al. The role of health systems in shaping vaccine decisions: insights from Italy, Mexico, the United Kingdom, and the United States. Vaccine. 2025;54:127134. doi: 10.1016/j.vaccine.2025.127134 Epub 20250416. PubMed PMID: 40245768. 40245768

[20] Robinson R, Nguyen E, Wright M, et al. Factors contributing to vaccine hesitancy and reduced vaccine confidence in rural underserved populations. Humanit Soc Sci Commun. 2022;9:416. doi: 10.1057/s41599-022-01439-3 Epub 20221124. PubMed PMID: 36466708; PubMed Central PMCID: PMC9702767. 36466708 PMC9702767

[21] Dube E, Laberge C, Guay M, et al. Vaccine hesitancy: an overview. Hum Vaccin Immunother. 2013;9:1763–1773. doi: 10.4161/hv.24657 Epub 20130412. PubMed PMID: 23584253; PubMed Central PMCID: PMC3906279. 23584253 PMC3906279

[22] Cadeddu C, Castagna C, Sapienza M, et al. Understanding the determinants of vaccine hesitancy and vaccine confidence among adolescents: a systematic review. Hum Vaccin Immunother. 2021;17:4470–4486. doi: 10.1080/21645515.2021.1961466 Epub 20210902. PubMed PMID: 34473589; PubMed Central PMCID: PMC8828162. 34473589 PMC8828162

[23] Deal A, Crawshaw AF, Carter J, et al. Defining drivers of under-immunization and vaccine hesitancy in refugee and migrant populations. J Travel Med. 2023;30. doi: 10.1093/jtm/taad084 PMC10481413 37335192

[24] Galagali PM, Kinikar AA, Kumar VS. Vaccine hesitancy: obstacles and challenges. Curr Pediatr Rep. 2022;10:241–248. doi: 10.1007/s40124-022-00278-9 Epub 20221008. PubMed PMID: 36245801; PubMed Central PMCID: PMC9546747. 36245801 PMC9546747

[25] WHO. Confidence and demand. WHO; 2025 [cited 2025 Jul 31]. Available from: https://www.who.int/teams/immunization-vaccines-and-biologicals/essential-programme-on-immunization/demand

[26] Dyda A, King C, Dey A, et al. A systematic review of studies that measure parental vaccine attitudes and beliefs in childhood vaccination. BMC Public Health. 2020;20:1253. doi: 10.1186/s12889-020-09327-8 Epub 2020/08/19. PubMed PMID: 32807124; PubMed Central PMCID: PMC7433363. 32807124 PMC7433363

[27] Smith LE, Amlôt R, Weinman J, et al. A systematic review of factors affecting vaccine uptake in young children. Vaccine. 2017;35:6059–6069. doi: 10.1016/j.vaccine.2017.09.046 28974409

[28] Larson HJ, Jarrett C, Eckersberger E, et al. Understanding vaccine hesitancy around vaccines and vaccination from a global perspective: a systematic review of published literature, 2007–2012. Vaccine. 2014;32:2150–2159. doi: 10.1016/j.vaccine.2014.01.081 Epub 20140302. PubMed PMID: 24598724. 24598724

[29] Schmid P, Rauber D, Betsch C, et al. Barriers of influenza vaccination intention and behavior - a systematic review of influenza vaccine hesitancy, 2005 – 2016. PLoS One. 2017;12:e0170550. doi: 10.1371/journal.pone.0170550 Epub 20170126. PubMed PMID: 28125629; PubMed Central PMCID: PMC5268454. 28125629 PMC5268454

[30] Lack A, Hiligsmann M, Bloem P, et al. Parent, provider and vaccinee preferences for HPV vaccination: a systematic review of discrete choice experiments. Vaccine. 2020;38:7226–7238. doi: 10.1016/j.vaccine.2020.08.078 Epub 20201003. PubMed PMID: 33023774. 33023774

[31] Everett JA, Faber NS, Crockett M. Preferences and beliefs in ingroup favoritism. Front Behav Neurosci. 2015;9:15. doi: 10.3389/fnbeh.2015.00015 Epub 20150213. PubMed PMID: 25762906; PubMed Central PMCID: PMC4327620. 25762906 PMC4327620

[32] Gyasi SF, Kumi W, Kwofie C. Factors influencing individual vaccine preferences for COVID-19 in the Sunyani municipality, Ghana: an observational study using discrete choice experiment analysis. Health Sci Rep. 2024;7:e2263. doi: 10.1002/hsr2.2263 Epub 20240723. PubMed PMID: 39050907; PubMed Central PMCID: PMC11265991. 39050907 PMC11265991

[33] Diks ME, Hiligsmann M, van der Putten IM. Vaccine preferences driving vaccine-decision making of different target groups: a systematic review of choice-based experiments. BMC Infect Dis. 2021;21:879. doi: 10.1186/s12879-021-06398-9 Epub 20210828. PubMed PMID: 34454441; PubMed Central PMCID: PMC8397865. 34454441 PMC8397865

[34] Zajonc R, Markus H. Affective and cognitive factors in preferences. J Consum Res. 1982;9:123–131. doi: 10.1086/208905

[35] Poulos C, Standaert B, Sloesen B, et al. Preferences for vaccines against children’s diarrheal illness among mothers in Poland and Hungary. Vaccine. 2018;36:6022–6029. doi: 10.1016/j.vaccine.2018.08.001 Epub 20180824. PubMed PMID: 30150163. 30150163

[36] Eilers R, de Melker HE, Veldwijk J, et al. Vaccine preferences and acceptance of older adults. Vaccine. 2017;35:2823–2830. doi: 10.1016/j.vaccine.2017.04.014 Epub 20170412. PubMed PMID: 28412075. 28412075

[37] Boger S, van Bergen I, Beaudart C, et al. Preference of young adults for COVID-19 vaccination in the United Kingdom: a discrete choice experiment. Expert Rev Pharmacoecon Outcomes Res. 2023;23:921–931. doi: 10.1080/14737167.2023.2223983 Epub 20230614. PubMed PMID: 37294709. 37294709

[38] Smith LE, Carter B. Parental preferences for a mandatory vaccination scheme in England: a discrete choice experiment. Lancet Reg Health Eur. 2022;16:100359. doi: 10.1016/j.lanepe.2022.100359 Epub 20220413. PubMed PMID: 35570849; PubMed Central PMCID: PMC9097614. 35570849 PMC9097614

[39] Yates JF, de Oliveira S. Culture and decision making. Organ Behav Hum Decis Process. 2016;136:106–118. doi: 10.1016/j.obhdp.2016.05.003 Epub 20160914. PubMed PMID: 32288179; PubMed Central PMCID: PMC7126161. 32288179 PMC7126161

[40] Michaels-Igbokwe C, MacDonald S, Currie GR. Individual preferences for child and adolescent vaccine attributes: a systematic review of the stated preference literature. Patient. 2017;10:687–700. doi: 10.1007/s40271-017-0244-x PubMed PMID: 28474295. 28474295

[41] Ngorsuraches S, Nawanukool K, Petcharamanee K, et al. Parents’ preferences and willingness-to-pay for human papilloma virus vaccines in Thailand. J Pharm Policy Pract. 2015;8:20. doi: 10.1186/s40545-015-0040-8 Epub 20150722. PubMed PMID: 26199734; PubMed Central PMCID: PMC4509725. 26199734 PMC4509725

[42] Alsuwaidi AR, Hammad HAA, Elbarazi I, et al. Vaccine hesitancy within the Muslim community: islamic faith and public health perspectives. Hum Vaccin Immunother. 2023;19:2190716. doi: 10.1080/21645515.2023.2190716 Epub 20230313. PubMed PMID: 36914409; PubMed Central PMCID: PMC10038058. 36914409 PMC10038058

[43] O’Kane C. Most Americans say they prefer a specific brand of vaccine – and what they’ve seen on social media influences their decision 2021. [cited 2021 Apr 30]. Available from: https://www.cbsnews.com/news/covid-19-vaccine-brand-preference/

[44] Amit AML, Pepito VC, Sumpaico-Tanchanco L, et al. COVID-19 vaccine brand hesitancy and other challenges to vaccination in the Philippines. PLOS Glob Public Health. 2022;2:e0000165. doi: 10.1371/journal.pgph.0000165 36962166 PMC10021706

[45] Kuo CT, Yu RR. Association of national identity and trust in government with COVID-19 vaccination and brand choice in Taiwan. J Community Health. 2024;49:967–976. doi: 10.1007/s10900-024-01347-4 Epub 20240324. PubMed PMID: 38522040. 38522040

[46] Ong AKS, Prasetyo YT, Lagura FC, et al. Young adult preference analysis on the attributes of COVID-19 vaccine in the Philippines: a conjoint analysis approach. Public Health Pract (Oxf). 2022;4:100300. doi: 10.1016/j.puhip.2022.100300 Epub 20220719. PubMed PMID: 35874794; PubMed Central PMCID: PMC9293378. 35874794 PMC9293378

[47] O’Kane C. Most Americans say they prefer a specific brand of vaccine – and what they’ve seen on social media influences their decision. 2021. https://www.cbsnews.com/news/covid-19-vaccine-brand-preference/.

[48] Noblit GW, Hare DR. Meta-ethnography: synthesizing qualitative studies. Newbury Park (CA): SAGE Publications, Inc.; 1988.

[49] France EF, Uny I, Ring N, et al. A methodological systematic review of meta-ethnography conduct to articulate the complex analytical phases. BMC Med Res Methodol. 2019;19:35. doi: 10.1186/s12874-019-0670-7 Epub 2019/02/20. PubMed PMID: 30777031; PubMed Central PMCID: PMC6380066. 30777031 PMC6380066

[50] Atkins S, Lewin S, Smith H, Engel M, Fretheim A, Volmink J. Conducting a meta-ethnography of qualitative literature: lessons learnt. BMC Med Res Methodol. 2008 Apr 16;8:21. doi: 10.1186/1471-2288-8-21. PMC2374791 18416812

[51] Sattar R, Lawton R, Panagioti M, et al. Meta-ethnography in healthcare research: a guide to using a meta-ethnographic approach for literature synthesis. BMC Health Serv Res. 2021;21:50. doi: 10.1186/s12913-020-06049-w Epub 20210108. PubMed PMID: 33419430; PubMed Central PMCID: PMC7796630. 33419430 PMC7796630

[52] Ahmed SK, Mohammed RA, Nashwan AJ, et al. Using thematic analysis in qualitative research. J Med Surg Public Health. 2025;6:100198. doi: 10.1016/j.glmedi.2025.100198

[53] Kotler P, Keller KL. A framework for marketing management. 6th ed. Boston: Pearson Education; 2016.

[54] Zheng B, Yan J, Wang X, et al. Using the ‘4Ps’ social marketing strategy to overcome vaccination hesitancy: COVID-19 vaccine coverage in a Chinese college as an example. Saudi Med J. 2023;44:560–596. doi: 10.15537/smj.2023.44.6.20220696 PubMed PMID: 37343992; PubMed Central PMCID: PMC10284222. 37343992 PMC10284222

[55] Bank W. World Bank income groups, 2023 2024. [cited 2025 Jun 16]. Available from: https://ourworldindata.org/grapher/world-bank-income-groups

[56] Rumetta J, Abdul-Hadi H, Lee YK. A qualitative study on parents’ reasons and recommendations for childhood vaccination refusal in Malaysia. J Infect Public Health. 2020;13:199–203. doi: 10.1016/j.jiph.2019.07.027 Epub 2019/08/23. PubMed PMID: 31431420. 31431420

[57] Wang LD, Lam WW, Wu JT, et al. Chinese immigrant parents’ vaccination decision making for children: a qualitative analysis. BMC Public Health. 2014;14:133. doi: 10.1186/1471-2458-14-133 Epub 2014/02/11. PubMed PMID: 24507384; PubMed Central PMCID: PMC3937074. 24507384 PMC3937074

[58] Bland M, Clear GM, Grogan A, et al. Mum’s the word: factors that influenced young adults’ participation in the New Zealand Meningococcal B immunisation programme. N Z Med J. 2009;122:30–38. Epub 2010/02/12. PubMed PMID: 20148042. 20148042

[59] Harmsen IA, Bos H, Ruiter RA, et al. Vaccination decision-making of immigrant parents in the Netherlands; a focus group study. BMC Public Health. 2015;15:1229. doi: 10.1186/s12889-015-2572-x Epub 2015/12/15. PubMed PMID: 26654538; PubMed Central PMCID: PMC4676170. 26654538 PMC4676170

[60] Lockyer B, Islam S, Rahman A, et al. Understanding COVID-19 misinformation and vaccine hesitancy in context: findings from a qualitative study involving citizens in Bradford, UK. Health Expect. 2021;24:1158–1167. doi: 10.1111/hex.13240 Epub 2021/05/05. PubMed PMID: 33942948; PubMed Central PMCID: PMC8239544. 33942948 PMC8239544

[61] Siu JY. Barriers to receiving human papillomavirus vaccination among female students in a university in Hong Kong. Cult Health Sex. 2013;15:1071–1084. doi: 10.1080/13691058.2013.807518 Epub 2013/07/06. PubMed PMID: 23826650. 23826650

[62] Newman PA, Woodford MR, Logie C. HIV vaccine acceptability and culturally appropriate dissemination among sexually diverse Aboriginal peoples in Canada. Glob Public Health. 2012;7:87–100. doi: 10.1080/17441692.2010.549139 Epub 2011/03/11. PubMed PMID: 21390966. 21390966

[63] Kajungu D, Muhoozi M, Stark J, et al. Vaccines safety and maternal knowledge for enhanced maternal immunization acceptability in rural Uganda: a qualitative study approach. PLoS One. 2020;15:e0243834. doi: 10.1371/journal.pone.0243834 Epub 2020/12/11. PubMed PMID: 33301495; PubMed Central PMCID: PMC7728220 Consulting respectively. They have no known competing financial interests or personal relationships that could have appeared to influence the work reported in this paper. This does not alter our adherence to PLoS One policies on sharing data and materials. 33301495 PMC7728220

[64] Glenn BA, Nonzee NJ, Tieu L, et al. Human papillomavirus (HPV) vaccination in the transition between adolescence and adulthood. Vaccine. 2021;39:3435–3444. doi: 10.1016/j.vaccine.2021.04.019 Epub 2021/05/17. PubMed PMID: 33992435. 33992435 PMC9063977

[65] Hopfer S, Clippard JR. College women’s HPV vaccine decision narratives. Qual Health Res. 2011;21:262–277. doi: 10.1177/1049732310383868 Epub 2010/09/16. PubMed PMID: 20841433. 20841433

[66] Katz ML, Reiter PL, Heaner S, et al. Acceptance of the HPV vaccine among women, parents, community leaders, and healthcare providers in Ohio Appalachia. Vaccine. 2009;27:3945–3952. doi: 10.1016/j.vaccine.2009.04.040 Epub 2009/04/25. PubMed PMID: 19389447; PubMed Central PMCID: PMC2700122. 19389447 PMC2700122

[67] Wilson R, Brown DR, Boothe MA, et al. Knowledge and acceptability of the HPV vaccine among ethnically diverse Black women. J Immigr Minor Health. 2013;15:747–757. doi: 10.1007/s10903-012-9749-5 Epub 2012/12/01. PubMed PMID: 23197180. 23197180

[68] Bair RM, Mays RM, Sturm LA, et al. Acceptability of the human papillomavirus vaccine among Latina mothers. J Pediatr Adolesc Gynecol. 2008;21:329–334. doi: 10.1016/j.jpag.2008.02.007 Epub 2008/12/10. PubMed PMID: 19064226. 19064226

[69] Yuen CY, Dodgson JE, Tarrant M. Perceptions of Hong Kong Chinese women toward influenza vaccination during pregnancy. Vaccine. 2016;34:33–40. doi: 10.1016/j.vaccine.2015.11.032 Epub 2015/12/01. PubMed PMID: 26616554. 26616554

[70] Huang W, Dove-Medows E, Shealey J, et al. COVID-19 vaccine attitudes among a majority Black sample in the southern US: public health implications from a qualitative study. BMC Public Health. 2023;23:88. doi: 10.1186/s12889-022-14905-z Epub 20230112. PubMed PMID: 36631819; PubMed Central PMCID: PMC9834032. 36631819 PMC9834032

[71] Knight KR, Duke MR, Carey CA, et al. COVID-19 testing and vaccine acceptability among homeless-experienced adults: qualitative data from two samples. J Gen Intern Med. 2022;37:823–829. doi: 10.1007/s11606-021-07161-1 Epub 20211026. PubMed PMID: 34704204; PubMed Central PMCID: PMC8547296. PMC8547296 34704204

[72] Jha SS, Paul B, Das R, et al. Contributing factors of willingness and hesitancy regarding acceptance of COVID-19 vaccine in primary care settings: a qualitative study in an eastern state of India. J Educ Health Promot. 2022;11:53. doi: 10.4103/jehp.jehp_363_21 PubMed PMID: 01679914-202211000-00052. 35372619 PMC8974975

[73] Sides E, Jones LF, Kamal A, et al. Attitudes towards coronavirus (COVID-19) vaccine and sources of information across diverse ethnic groups in the UK: a qualitative study from June to October 2020. BMJ Open. 2022;12:e060992. doi: 10.1136/bmjopen-2022-060992 Epub 20220901. PubMed PMID: 36581971; PubMed Central PMCID: PMC9437733. PMC9437733 36581971

[74] Steffens MS, Bullivant B, King C, et al. “I’m scared that if I have the vaccine, it’s going to make my lung condition worse, not better.” COVID-19 vaccine acceptance in adults with underlying health conditions - a qualitative investigation. Vaccine: X. 2022;12:100243. doi: 10.1016/j.jvacx.2022.100243 Epub 20221124. PubMed PMID: 36447620; PubMed Central PMCID: PMC9686055. 36447620 PMC9686055

[75] Dubé E, Labbé F, Malo B, et al. “I don’t think there’s a point for me to discuss it with my patients”: exploring health care providers’ views and behaviours regarding COVID-19 vaccination. Hum Vaccin Immunother. 2022;18:2088970. doi: 10.1080/21645515.2022.2088970 Epub 20220629. PubMed PMID: 35767434; PubMed Central PMCID: PMC9621068. 35767434 PMC9621068

[76] Newman PA, Seiden DS, Roberts KJ, et al. A small dose of HIV? HIV vaccine mental models and risk communication. Health Educ Behav. 2009;36:321–333. doi: 10.1177/1090198107305078 Epub 2007/11/23. PubMed PMID: 18032589. 18032589

[77] Berman M, Dube E, Quach C. Exploring the acceptability of the available pneumococcal conjugate vaccines in Canadian health care professionals and immunization experts. Vaccine. 2017;35:3326–3332. doi: 10.1016/j.vaccine.2017.04.083 Epub 20170510. PubMed PMID: 28501455. 28501455

[78] Visser O, Hautvast JL, van der Velden K, et al. Intention to accept pertussis vaccination for cocooning: a qualitative study of the determinants. PLoS One. 2016;11:e0155861. doi: 10.1371/journal.pone.0155861 Epub 20160602. PubMed PMID: 27253386; PubMed Central PMCID: PMC4890858. 27253386 PMC4890858

[79] Paterson P, Chantler T, Larson HJ. Reasons for non-vaccination: parental vaccine hesitancy and the childhood influenza vaccination school pilot programme in England. Vaccine. 2018;36:5397–5401. doi: 10.1016/j.vaccine.2017.08.016 Epub 20170814. PubMed PMID: 28818568. 28818568

[80] Telford R, Rogers A. What influences elderly peoples’ decisions about whether to accept the influenza vaccination? A qualitative study. Health Educ Res. 2003;18:743–753. doi: 10.1093/her/cyf059 PubMed PMID: 14654506. 14654506

[81] Evans MR, Prout H, Prior L, et al. A qualitative study of lay beliefs about influenza immunisation in older people. Br J Gen Pract. 2007;57:352–358. Epub 2007/05/17. PubMed PMID: 17504584; PubMed Central PMCID: PMC2047008. 17504584 PMC2047008

[82] Ambali RT, John-Akinola YO, Oluwasanu MM. Indepth interviews’ on acceptability and concerns for human papilloma virus vaccine uptake among mothers of adolescent girls in community settings in Ibadan, Nigeria. J Cancer Educ. 2022;37:748–754. doi: 10.1007/s13187-020-01876-1 Epub 20200916. PubMed PMID: 32939737. 32939737

[83] McComb E, Ramsden V, Olatunbosun O, et al. Knowledge, attitudes and barriers to human papillomavirus (HPV) vaccine uptake among an immigrant and refugee catch-up group in a western Canadian province. J Immigr Minor Health. 2018;20:1424–1428. doi: 10.1007/s10903-018-0709-6 PubMed PMID: 29445898. 29445898

[84] Krawczyk A, Perez S, King L, et al. Parents’ decision-making about the human papillomavirus vaccine for their daughters: II. Qualitative results. Hum Vaccin Immunother. 2015;11:330–336. doi: 10.4161/21645515.2014.980708 PubMed PMID: 25692507; PubMed Central PMCID: PMC4514412. 25692507 PMC4514412

[85] Kobetz E, Menard J, Hazan G, et al. Perceptions of HPV and cervical cancer among Haitian immigrant women: implications for vaccine acceptability. Educ Health (Abingdon). 2011;24:479. Epub 2012/01/24. PubMed PMID: 22267344. doi: 10.4103/1357-6283.101428 22267344

[86] Burghouts J, Del Nogal B, Uriepero A, et al. Childhood vaccine acceptance and refusal among Warao Amerindian caregivers in Venezuela; a qualitative approach. PLoS One. 2017;12:e0170227. doi: 10.1371/journal.pone.0170227 28107501 PMC5249092

[87] Jackson C, Bedford H, Cheater FM, et al. Needles, jabs and jags: a qualitative exploration of barriers and facilitators to child and adult immunisation uptake among Gypsies, Travellers and Roma. BMC Public Health. 2017;17:254. doi: 10.1186/s12889-017-4178-y Epub 20170314. PubMed PMID: 28288596; PubMed Central PMCID: PMC5348901. 28288596 PMC5348901

[88] McIntyre A, Zecevic A, Diachun L. Influenza vaccinations: older adults’ decision-making process. Can J Aging. 2014;33:92–98. doi: 10.1017/S0714980813000640 Epub 20131202. PubMed PMID: 24289886. 24289886

[89] Xiong S, Kasouaher MY, Vue B, et al. “We will do whatever it takes”: understanding socioecological level influences on Hmong-American adolescents and parents’ perceptions of the human papillomavirus vaccine. J Cancer Educ. 2021;37:1893–1901. doi: 10.1007/s13187-021-02057-4 34164765 PMC8221556

[90] Katahoire ARP, Wani JAMPH, Murokora DMD, et al. Acceptability of HPV vaccine among young adolescent girls in Uganda: young people’s perspectives count. Int J Child Adolesc Health. 2013;6:211–219. PubMed PMID: 1625518600.

[91] McClelland A, Liamputtong P. Knowledge and acceptance of human papillomavirus vaccination: perspectives of young Australians living in Melbourne, Australia. Sex Health. 2006;3:95–101. doi: 10.1071/sh05035 PubMed PMID: 16800395. 16800395

[92] Eilers R, Krabbe PF, de Melker HE. Motives of Dutch persons aged 50 years and older to accept vaccination: a qualitative study. BMC Public Health. 2015;15:493. doi: 10.1186/s12889-015-1825-z Epub 20150516. PubMed PMID: 25981624; PubMed Central PMCID: PMC4446004. 25981624 PMC4446004

[93] Meharry PM, Colson ER, Grizas AP, et al. Reasons why women accept or reject the trivalent inactivated influenza vaccine (TIV) during pregnancy. Matern Child Health J. 2013;17:156–164. doi: 10.1007/s10995-012-0957-3 PubMed PMID: 22367067. 22367067

[94] Fernandez-Pineda M, Cianelli R, Villegas N, et al. Salient factors among Hispanic parents in South Florida rural communities for vaccinating their children against human papillomavirus. J Pediatr Nurs. 2020;54:24–33. doi: 10.1016/j.pedn.2020.05.016 32521437 PMC7484143

[95] Morales-Campos DY, Snipes SA, Villarreal EK, et al. Cervical cancer, human papillomavirus (HPV), and HPV vaccination: exploring gendered perspectives, knowledge, attitudes, and cultural taboos among Mexican American adults. Ethn Health. 2021;26:206–224. doi: 10.1080/13557858.2018.1494821 Epub 20180712. PubMed PMID: 29998738; PubMed Central PMCID: PMC6330137. 29998738 PMC6330137

[96] Mitchell KR, Erio T, Whitworth HS, et al. Does the number of doses matter? A qualitative study of HPV vaccination acceptability nested in a dose reduction trial in Tanzania. Tumour Virus Res. 2021;12:200217. doi: 10.1016/j.tvr.2021.200217 Epub 20210526. PubMed PMID: 34051389; PubMed Central PMCID: PMC8233223. 34051389 PMC8233223

[97] Singh S, Sahu D, Agrawal A, et al. Barriers and opportunities for improving childhood immunization coverage in slums: a qualitative study. Prev Med Rep. 2019;14:100858. doi: 10.1016/j.pmedr.2019.100858 Epub 20190328. PubMed PMID: 30997325; PubMed Central PMCID: PMC6453822. 30997325 PMC6453822

[98] Winslade CG, Heffernan CM, Atchison CJ. Experiences and perspectives of mothers of the pertussis vaccination programme in London. Public Health. 2017;146:10–14. doi: 10.1016/j.puhe.2016.12.018 Epub 20170201. PubMed PMID: 28404461. 28404461

[99] Turiho AK, Okello ES, Muhwezi WW, et al. Perceptions of human papillomavirus vaccination of adolescent schoolgirls in Western Uganda and their implications for acceptability of HPV vaccination: a qualitative study. BMC Res Notes. 2017;10:431. doi: 10.1186/s13104-017-2749-8 Epub 20170830. PubMed PMID: 28854964; PubMed Central PMCID: PMC5577844. 28854964 PMC5577844

[100] Madhivanan P, Krupp K, Yashodha MN, et al. Attitudes toward HPV vaccination among parents of adolescent girls in Mysore, India. Vaccine. 2009;27:5203–5208. doi: 10.1016/j.vaccine.2009.06.073 19596420

[101] Long S, Wu J, Wang S, et al. Changes of factors associated with vaccine hesitancy in Chinese residents: a qualitative study. Front Public Health. 2022;10:929407. doi: 10.3389/fpubh.2022.929407 Epub 20220920. PubMed PMID: 36203693; PubMed Central PMCID: PMC9530596. 36203693 PMC9530596

[102] Biezen R, Grando D, Mazza D, et al. Why do we not want to recommend influenza vaccination to young children? A qualitative study of Australian parents and primary care providers. Vaccine. 2018;36:859–865. doi: 10.1016/j.vaccine.2017.12.066 Epub 20180105. PubMed PMID: 29310901. 29310901

[103] Flood EM, Block SL, Hall MC, et al. Children’s perceptions of influenza illness and preferences for influenza vaccine. J Pediatr Health Care. 2011;25:171–179. doi: 10.1016/j.pedhc.2010.04.007 21514492

[104] Sun X, Huang Z, Wagner AL, et al. The role of severity perceptions and beliefs in natural infections in Shanghai parents’ vaccine decision-making: a qualitative study. BMC Public Health. 2018;18:813. doi: 10.1186/s12889-018-5734-9 Epub 20180628. PubMed PMID: 29954371; PubMed Central PMCID: PMC6025737. 29954371 PMC6025737

[105] Romijnders K, van Seventer SL, Scheltema M, et al. A deliberate choice? Exploring factors related to informed decision-making about childhood vaccination among acceptors, refusers, and partial acceptors. Vaccine. 2019;37:5637–5644. doi: 10.1016/j.vaccine.2019.07.060 Epub 2019/08/07. PubMed PMID: 31383488. 31383488

[106] Cover JK, Nghi NQ, LaMontagne DS, et al. Acceptance patterns and decision-making for human papillomavirus vaccination among parents in Vietnam: an in-depth qualitative study post-vaccination. BMC Public Health. 2012;12:629. doi: 10.1186/1471-2458-12-629 22877158 PMC3437216

[107] Fadda M, Depping MK, Schulz PJ. Addressing issues of vaccination literacy and psychological empowerment in the measles-mumps-rubella (MMR) vaccination decision-making: a qualitative study. BMC Public Health. 2015;15:836. doi: 10.1186/s12889-015-2200-9 Epub 20150902. PubMed PMID: 26328551; PubMed Central PMCID: PMC4556054. 26328551 PMC4556054

[108] Westrick SC, Hohmann LA, McFarland SJ, et al. Parental acceptance of human papillomavirus vaccinations and community pharmacies as vaccination settings: a qualitative study in Alabama. Papillomavirus Res. 2017;3:24–29. doi: 10.1016/j.pvr.2016.12.003 Epub 20161221. PubMed PMID: 28720453; PubMed Central PMCID: PMC5883249. 28720453 PMC5883249

[109] Fadda M, Suggs LS, Albanese E. Willingness to vaccinate against COVID-19: a qualitative study involving older adults from southern Switzerland. Vaccine: X. 2021;8:100108. doi: 10.1016/j.jvacx.2021.100108 Epub 20210715. PubMed PMID: 34308329; PubMed Central PMCID: PMC8279929. 34308329 PMC8279929

[110] Jalloh MF, Jalloh MB, Albert A, et al. Perceptions and acceptability of an experimental Ebola vaccine among health care workers, frontline staff, and the general public during the 2014–2015 Ebola outbreak in Sierra Leone. Vaccine. 2019;37:1495–1502. doi: 10.1016/j.vaccine.2019.01.046 Epub 2019/02/14. PubMed PMID: 30755367; PubMed Central PMCID: PMC7393388. 30755367 PMC7393388

[111] Gottvall M, Stenhammar C, Grandahl M. Parents’ views of including young boys in the Swedish national school-based HPV vaccination programme: a qualitative study. BMJ Open. 2017;7:e014255. doi: 10.1136/bmjopen-2016-014255 Epub 20170228. PubMed PMID: 28246143; PubMed Central PMCID: PMC5337740. PMC5337740 28246143

[112] Deal A, Hayward SE, Huda M, et al. Strategies and action points to ensure equitable uptake of COVID-19 vaccinations: a national qualitative interview study to explore the views of undocumented migrants, asylum seekers, and refugees. J Migr Health. 2021;4:100050. doi: 10.1016/j.jmh.2021.100050 Epub 20210527. PubMed PMID: 34075367; PubMed Central PMCID: PMC8154190. 34075367 PMC8154190

[113] Siu JY. Perceptions of and barriers to vaccinating daughters against human papillomavirus (HPV) among mothers in Hong Kong. BMC Womens Health. 2014;14:73. doi: 10.1186/1472-6874-14-73 Epub 20140602. PubMed PMID: 24890226; PubMed Central PMCID: PMC4049476. 24890226 PMC4049476

[114] Padmawati RS, Heywood A, Sitaresmi MN, et al. Religious and community leaders’ acceptance of rotavirus vaccine introduction in Yogyakarta, Indonesia: a qualitative study. BMC Public Health. 2019;19:368. doi: 10.1186/s12889-019-6706-4 Epub 20190403. PubMed PMID: 30943929; PubMed Central PMCID: PMC6446267. 30943929 PMC6446267

[115] Kanagat N, Krudwig K, Wilkins KA, et al. Health care worker preferences and perspectives on doses per container for 2 lyophilized vaccines in Senegal, Vietnam, and Zambia. Glob Health Sci Pract. 2020;8:680–688. doi: 10.9745/ghsp-d-20-00112 Epub 20201223. PubMed PMID: 33361235; PubMed Central PMCID: PMC7784065. 33361235 PMC7784065

[116] Fleming T, Valleriani J, Ng C, et al. Acceptability of a hypothetical preventative HIV vaccine among people who use drugs in Vancouver, Canada. BMC Public Health. 2020;20:1081. doi: 10.1186/s12889-020-09202-6 Epub 2020/07/11. PubMed PMID: 32646390; PubMed Central PMCID: PMC7350753. 32646390 PMC7350753

[117] Bell S, Edelstein M, Zatonski M, et al. ‘I don’t think anybody explained to me how it works’: qualitative study exploring vaccination and primary health service access and uptake amongst Polish and Romanian communities in England. BMJ Open. 2019;9:e028228. doi: 10.1136/bmjopen-2018-028228 Epub 2019/07/11. PubMed PMID: 31289079; PubMed Central PMCID: PMC6615777. PMC6615777 31289079

[118] Sampson R, Wong L, Macvicar R. Parental reasons for non-uptake of influenza vaccination in young at-risk groups: a qualitative study. Br J Gen Pract. 2011;61:e386–91. doi: 10.3399/bjgp11X583155 PubMed PMID: 21722445; PubMed Central PMCID: PMC3123500. 21722445 PMC3123500

[119] Simas C, Larson HJ, Paterson P. “Saint Google, now we have information!”: a qualitative study on narratives of trust and attitudes towards maternal vaccination in Mexico City and Toluca. BMC Public Health. 2021;21:1170. doi: 10.1186/s12889-021-11184-y Epub 20210618. PubMed PMID: 34144703; PubMed Central PMCID: PMC8212502. 34144703 PMC8212502

[120] Simas C, Larson HJ, Paterson P. ”Those who do not vaccinate don’t love themselves, or anyone else”: a qualitative study of views and attitudes of urban pregnant women towards maternal immunisation in Panama. BMJ Open. 2021;11:e044903. doi: 10.1136/bmjopen-2020-044903 Epub 20210820. PubMed PMID: 34417210; PubMed Central PMCID: PMC8381308. PMC8381308 34417210

[121] Kwong EW, Pang SM, Choi PP, et al. Influenza vaccine preference and uptake among older people in nine countries. J Adv Nurs. 2010;66:2297–2308. doi: 10.1111/j.1365-2648.2010.05397.x Epub 20100816. PubMed PMID: 20722815. 20722815

[122] Pugliese-Garcia M, Heyerdahl LW, Mwamba C, et al. Factors influencing vaccine acceptance and hesitancy in three informal settlements in Lusaka, Zambia. Vaccine. 2018;36:5617–5624. doi: 10.1016/j.vaccine.2018.07.042 Epub 20180804. PubMed PMID: 30087047; PubMed Central PMCID: PMC6143480. 30087047 PMC6143480

[123] Remes P, Selestine V, Changalucha J, et al. A qualitative study of HPV vaccine acceptability among health workers, teachers, parents, female pupils, and religious leaders in Northwest Tanzania. Vaccine. 2012;30:5363–5367. doi: 10.1016/j.vaccine.2012.06.025 Epub 2012/06/27. PubMed PMID: 22732428; PubMed Central PMCID: PMC3409375. 22732428 PMC3409375

[124] Colmegna I, Valerio V, Gosselin-Boucher V, et al. Barriers and facilitators to influenza and pneumococcal vaccine hesitancy in rheumatoid arthritis: a qualitative study. Rheumatology. 2021;60:5257–5270. doi: 10.1093/rheumatology/keab471 34086876

[125] Ramanadhan S, Fontanet C, Teixeira M, et al. Exploring attitudes of adolescents and caregivers towards community-based delivery of the HPV vaccine: a qualitative study. BMC Public Health. 2020;20:1531. doi: 10.1186/s12889-020-09632-2 Epub 20201009. PubMed PMID: 33036585; PubMed Central PMCID: PMC7547455. 33036585 PMC7547455

[126] Reiter PL, Oldach BR, Randle KE, et al. Acceptability of HPV vaccine for males and preferences for future education programs among Appalachian residents. Am J Mens Health. 2014;8:167–174. doi: 10.1177/1557988313505319 Epub 2013/10/03. PubMed PMID: 24085197; PubMed Central PMCID: PMC3987900. 24085197 PMC3987900

[127] Elbarazi I, Yacoub M, Reyad OA, et al. Exploring enablers and barriers toward COVID-19 vaccine acceptance among Arabs: a qualitative study. Int J Disaster Risk Reduct. 2022;82:103304. doi: 10.1016/j.ijdrr.2022.103304 Epub 20220929. PubMed PMID: 36193257; PubMed Central PMCID: PMC9519527. 36193257 PMC9519527

[128] Shah P, Shetty V, Ganesh M, et al. Challenges to human papillomavirus vaccine acceptability among women in South India: an exploratory study. Am J Trop Med Hyg. 2021;105:966–973. doi: 10.4269/ajtmh.20-1650 Epub 20210809. PubMed PMID: 34370698; PubMed Central PMCID: PMC8592141. 34370698 PMC8592141

[129] Tibbels NJ, Dosso A, Fordham C, et al. “On the last day of the last month, I will go”: a qualitative exploration of COVID-19 vaccine confidence among Ivoirian adults. Vaccine. 2022;40:2028–2035. doi: 10.1016/j.vaccine.2022.02.032 35181151 PMC8831134

[130] Paul P, Tanner AE, Gravitt PE, et al. Acceptability of HPV vaccine implementation among parents in India. Health Care Women Int. 2014;35:1148–1161. doi: 10.1080/07399332.2012.740115 Epub 20130423. PubMed PMID: 23611111; PubMed Central PMCID: PMC4503242. 23611111 PMC4503242

[131] Ganczak M, Bielecki K, Drozd-Dabrowska M, et al. Vaccination concerns, beliefs and practices among Ukrainian migrants in Poland: a qualitative study. BMC Public Health. 2021;21:93. doi: 10.1186/s12889-020-10105-9 Epub 20210107. PubMed PMID: 33413287; PubMed Central PMCID: PMC7789884. 33413287 PMC7789884

[132] Bullivant B, Bolsewicz KT, King C, et al. COVID-19 vaccination acceptance among older adults: a qualitative study in New South Wales, Australia. Public Health Pract (Oxf). 2023;5:100349. doi: 10.1016/j.puhip.2022.100349 Epub 20221210. PubMed PMID: 36532098; PubMed Central PMCID: PMC9737511. 36532098 PMC9737511

[133] Duong MC, Nguyen HT, Duong M. Evaluating COVID-19 vaccine hesitancy: a qualitative study from Vietnam. Diabetes Metab Syndr. 2022;16:102363. doi: 10.1016/j.dsx.2021.102363 Epub 20211209. PubMed PMID: 34922216; PubMed Central PMCID: PMC8656146. 34922216 PMC8656146

[134] Yoon S, Goh H, Matchar D, et al. Multifactorial influences underpinning a decision on COVID-19 vaccination among healthcare workers: a qualitative analysis. Hum Vaccin Immunother. 2022;18:2085469. doi: 10.1080/21645515.2022.2085469 Epub 20220610. PubMed PMID: 35687802; PubMed Central PMCID: PMC9621075. 35687802 PMC9621075

[135] Balasuriya L, Santilli A, Morone J, et al. COVID-19 vaccine acceptance and access among Black and Latinx communities. JAMA Netw Open. 2021;4:e2128575. doi: 10.1001/jamanetworkopen.2021.28575 Epub 20211001. PubMed PMID: 34643719; PubMed Central PMCID: PMC8515205. 34643719 PMC8515205

[136] Giduthuri JG, Purohit V, Kudale A, et al. Antenatal influenza vaccination in urban Pune, India: clinician and community stakeholders’ awareness, priorities, and practices. Hum Vaccin Immunother. 2021;17:1211–1222. doi: 10.1080/21645515.2020.1806670 Epub 20200923. PubMed PMID: 32966146; PubMed Central PMCID: PMC8018408. 32966146 PMC8018408

[137] Carson SL, Casillas A, Castellon-Lopez Y, et al. COVID-19 vaccine decision-making factors in racial and ethnic minority communities in Los Angeles, California. JAMA Netw Open. 2021;4:e2127582. doi: 10.1001/jamanetworkopen.2021.27582 Epub 20210901. PubMed PMID: 34591103; PubMed Central PMCID: PMC8485164. 34591103 PMC8485164

[138] Tuckerman JL, Kaufman J, Danchin M, et al. Influenza vaccination: a qualitative study of practice level barriers from medical practitioners caring for children with special risk medical conditions. Vaccine. 2020;38:7806–7814. doi: 10.1016/j.vaccine.2020.10.020 Epub 20201023. PubMed PMID: 33164803. 33164803

[139] Sim JA, Ulanika AA, Katikireddi SV, et al. ‘Out of two bad choices, I took the slightly better one’: vaccination dilemmas for Scottish and Polish migrant women during the H1N1 influenza pandemic. Public Health. 2011;125:505–511. doi: 10.1016/j.puhe.2011.05.005 Epub 2011/08/02. PubMed PMID: 21802701. 21802701

[140] Ko LK, Taylor VM, Mohamed FB, et al. “We brought our culture here with us”: a qualitative study of perceptions of HPV vaccine and vaccine uptake among East African immigrant mothers. Papillomavirus Res. 2019;7:21–25. doi: 10.1016/j.pvr.2018.12.003 30594650 PMC6319298

[141] Marin-Cos A, Marban-Castro E, Nedic I, et al. “Maternal vaccination greatly depends on your trust in the healthcare system”: a qualitative study on the acceptability of maternal vaccines among pregnant women and healthcare workers in Barcelona, Spain. Vaccines (Basel). 2022;10:2015. doi: 10.3390/vaccines10122015 Epub 20221125. PubMed PMID: 36560425; PubMed Central PMCID: PMC9783547. 36560425 PMC9783547

[142] Benin AL, Wisler-Scher DJ, Colson E, et al. Qualitative analysis of mothers’ decision-making about vaccines for infants: the importance of trust. Pediatrics. 2006;117:1532–1541. doi: 10.1542/peds.2005-1728 PubMed PMID: 16651306. 16651306

[143] Waller J, Marlow LA, Wardle J. Mothers’ attitudes towards preventing cervical cancer through human papillomavirus vaccination: a qualitative study. Cancer Epidemiol Biomarker Prev. 2006;15:1257–1261. doi: 10.1158/1055-9965.Epi-06-0041 PubMed PMID: 16835320. 16835320

[144] Collins J, Alona I, Tooher R, et al. Increased awareness and health care provider endorsement is required to encourage pregnant women to be vaccinated. Hum Vaccin Immunother. 2014;10:2922–2929. doi: 10.4161/21645515.2014.971606 Epub 20141121. PubMed PMID: 25483464; PubMed Central PMCID: PMC5443072. 25483464 PMC5443072

[145] Enkel SL, Attwell K, Snelling TL, et al. ‘Hesitant compliers’: qualitative analysis of concerned fully-vaccinating parents. Vaccine. 2018;36:6459–6463. doi: 10.1016/j.vaccine.2017.09.088 Epub 20171012. PubMed PMID: 29031695. 29031695

[146] Karafillakis E, Paterson P, Larson HJ. ‘My primary purpose is to protect the unborn child’: understanding pregnant women’s perceptions of maternal vaccination and vaccine trials in Europe. Vaccine. 2021;39:5673–5679. doi: 10.1016/j.vaccine.2021.07.099 Epub 20210818. PubMed PMID: 34419304. 34419304

[147] Tarrant M, Gregory D. Exploring childhood immunization uptake with first nations mothers in north-western Ontario, Canada. J Adv Nurs. 2003;41:63–72. doi: 10.1046/j.1365-2648.2003.02507.x PubMed PMID: 12519289. 12519289

[148] Rubens-Augustson T, Wilson LA, Murphy MS, et al. Healthcare provider perspectives on the uptake of the human papillomavirus vaccine among newcomers to Canada: a qualitative study. Hum Vaccin Immunother. 2019;15:1697–1707. doi: 10.1080/21645515.2018.1539604 Epub 20181105. PubMed PMID: 30352005; PubMed Central PMCID: PMC6746509. 30352005 PMC6746509

[149] Charania NA, Watson DG, Turner NM. Perceptions of caregivers and providers regarding the potential introduction of the varicella vaccine to the childhood immunisation schedule in New Zealand: a qualitative exploratory study. J Paediatr Child Health. 2018;54:28–35. doi: 10.1111/jpc.13661 Epub 20170809. PubMed PMID: 28795455. 28795455

[150] Ecker F, Kutalek R. ‘I’m not an anti-vaxer!’-vaccine hesitancy among physicians: a qualitative study. Eur J Public Health. 2021;31:1157–1163. doi: 10.1093/eurpub/ckab174 PubMed PMID: 34580713; PubMed Central PMCID: PMC8675240. 34580713 PMC8675240

[151] Kristensen DD, Bartholomew K, Villadiego S, et al. What vaccine product attributes do immunization program stakeholders value? Results from interviews in six low- and middle-income countries. Vaccine. 2016;34:6236–6242. doi: 10.1016/j.vaccine.2016.10.057 Epub 20161108. PubMed PMID: 27836438. 27836438

[152] Wheelock A, Parand A, Rigole B, et al. Socio-psychological factors driving adult vaccination: a qualitative study. PLoS One. 2014;9:e113503. doi: 10.1371/journal.pone.0113503 Epub 20141209. PubMed PMID: 25490542; PubMed Central PMCID: PMC4260791. 25490542 PMC4260791

[153] Jalilian H, Amraei M, Javanshir E, et al. Ethical considerations of the vaccine development process and vaccination: a scoping review. BMC Health Serv Res. 2023;23:255. doi: 10.1186/s12913-023-09237-6 Epub 20230314. PubMed PMID: 36918888; PubMed Central PMCID: PMC10013982. 36918888 PMC10013982

[154] Igarashi A, Nakano Y, Yoneyama-Hirozane M. Public preferences and willingness to accept a hypothetical vaccine to prevent a pandemic in Japan: a conjoint analysis. Expert Rev Vaccines. 2022;21:241–248. doi: 10.1080/14760584.2022.2016402 Epub 2022/01/26. PubMed PMID: 35073824. 35073824

[155] Rodrigues CMC, Plotkin SA. Impact of vaccines; health, economic and social perspectives. Front Microbiol. 2020;11:1526. doi: 10.3389/fmicb.2020.01526 Epub 20200714. PubMed PMID: 32760367; PubMed Central PMCID: PMC7371956. 32760367 PMC7371956

[156] Capurro G, Maier R, Tustin J, et al. “They’re trying to bribe you and taking away your freedoms”: COVID-19 vaccine hesitancy in communities with traditionally low vaccination rates. Vaccine. 2022;40:7280–7287. doi: 10.1016/j.vaccine.2022.10.058 36334965 PMC9618440

[157] Orhierhor M, Rubincam C, Greyson D, et al. New mothers’ key questions about child vaccinations from pregnancy through toddlerhood: evidence from a qualitative longitudinal study in Victoria, British Columbia. SSM - Qualitative Res Health. 2023;3:100229. doi: 10.1016/j.ssmqr.2023.100229

[158] Fatima S, Zafar A, Afzal H, et al. COVID-19 infection among vaccinated and unvaccinated: does it make any difference? PLoS One. 2022;17:e0270485. doi: 10.1371/journal.pone.0270485 Epub 20220715. PubMed PMID: 35839210; PubMed Central PMCID: PMC9286242. 35839210 PMC9286242

[159] Whittaker R, Bråthen Kristofferson A, Valcarcel Salamanca B, et al. Length of hospital stay and risk of intensive care admission and in-hospital death among COVID-19 patients in Norway: a register-based cohort study comparing patients fully vaccinated with an mRNA vaccine to unvaccinated patients. Clin Microbiol Infect. 2022;28:871–878. doi: 10.1016/j.cmi.2022.01.033 Epub 20220225. PubMed PMID: 35219807; PubMed Central PMCID: PMC8872711. 35219807 PMC8872711

[160] Nuwarda RF, Ramzan I, Weekes L, et al. Vaccine hesitancy: contemporary issues and historical background. Vaccines (Basel). 2022;10:1595. doi: 10.3390/vaccines10101595 Epub 20220922. PubMed PMID: 36298459; PubMed Central PMCID: PMC9612044. 36298459 PMC9612044

[161] Adhikari B, Yeong Cheah P, von Seidlein L. Trust is the common denominator for COVID-19 vaccine acceptance: a literature review. Vaccine: X. 2022;12:100213. doi: 10.1016/j.jvacx.2022.100213 Epub 20220929. PubMed PMID: 36217424; PubMed Central PMCID: PMC9536059. 36217424 PMC9536059

[162] Murele B, Vaz R, Gasasira A, et al. Vaccine perception among acceptors and non-acceptors in Sokoto State, Nigeria. Vaccine. 2014;32:3323–3327. doi: 10.1016/j.vaccine.2014.03.050 Epub 2014/04/10. PubMed PMID: 24713368. 24713368

[163] James EK, Bokemper SE, Gerber AS, et al. Persuasive messaging to increase COVID-19 vaccine uptake intentions. Vaccine. 2021;39:7158–7165. doi: 10.1016/j.vaccine.2021.10.039 Epub 20211022. PubMed PMID: 34774363; PubMed Central PMCID: PMC8531257. 34774363 PMC8531257

[164] Guzman-Holst A, DeAntonio R, Prado-Cohrs D, et al. Barriers to vaccination in Latin America: a systematic literature review. Vaccine. 2020;38:470–481. doi: 10.1016/j.vaccine.2019.10.088 Epub 2019/11/27. PubMed PMID: 31767469. 31767469

[165] Dahie HA, Mohamoud JH, Adam MH, et al. COVID-19 vaccine coverage and potential drivers of vaccine uptake among healthcare workers in Somalia: a cross-sectional study. Vaccines (Basel). 2022;10:1116. doi: 10.3390/vaccines10071116 Epub 20220713. PubMed PMID: 35891280; PubMed Central PMCID: PMC9318518. 35891280 PMC9318518

[166] Larson HJ, Broniatowski DA. Volatility of vaccine confidence. Science. 2021;371:1289. doi: 10.1126/science.abi6488 Epub 2021/03/27. PubMed PMID: 33766861. 33766861

[167] Wachinger J, Renosa MDC, Endoma V, et al. Routines, disruptions, revised decisions: a biographical analysis of vaccination trajectories among Filipino caregivers. Vaccine. 2024;42:126095. doi: 10.1016/j.vaccine.2024.06.062 Epub 20240706. PubMed PMID: 38972765. 38972765

[168] Renosa MDC, Wachinger J, Barnighausen K, et al. Misinformation, infighting, backlash, and an ‘endless’ recovery; policymakers recount challenges and mitigating measures after a vaccine scare in the Philippines. Glob Health Action. 2022;15:2077536. doi: 10.1080/16549716.2022.2077536 PubMed PMID: 35930464; PubMed Central PMCID: PMC9359158. 35930464 PMC9359158

[169] Rahangdale L, Mungo C, O’Connor S, et al. Human papillomavirus vaccination and cervical cancer risk. BMJ. 2022;379:e070115. doi: 10.1136/bmj-2022-070115 Epub 20221215. PubMed PMID: 36521855. 36521855

[170] Ifeanyi C, Okechukwu E, Tosin O, et al. Assessing the determinants of uptake and hesitancy in accessing COVID 19 vaccines in Nigeria: a scoping review. Front Health Serv. 2025;5:1609418. doi: 10.3389/frhs.2025.1609418 Epub 20250904. PubMed PMID: 40980413; PubMed Central PMCID: PMC12443694. 40980413 PMC12443694

[171] Vardavas C, Nikitara K, Aslanoglou K, et al. Social determinants of health and vaccine uptake during the first wave of the COVID-19 pandemic: a systematic review. Prev Med Rep. 2023;35:102319. doi: 10.1016/j.pmedr.2023.102319 37564118 PMC10410576

