# Supplementary material for: Vaccine preferences and their role for vaccine confidence and uptake: a meta-ethnography
Source: Glob Health Action. 2026 Feb 13;19(1):2588846. doi: 10.1080/16549716.2025.2588846 (PMC12912219; doi:10.1080/16549716.2025.2588846)
Supplement: Supplementary Table 3_COREQ.pdf [file ZGHA_A_2588846_SM7904.pdf]

Supplementary Table 3. Quality Assessment of Final Included Studies

Reporting criteria			N (%) (n=97)	Study No.
**Domain 1: Research team and reflexivity**	Personal Characteristics	Interviewer/ facilitator	63 (64.94%)	2, 3, 4, 5, 7, 10, 12, 14, 15, 16, 17, 19, 21, 22, 24, 25, 26, 29, 32, 33, 34, 35, 36, 37, 39, 41, 42, 43, 48, 49, 50, 51, 52, 53, 55, 57, 58, 59, 60, 62, 63, 64, 65, 67, 68, 69, 70, 71, 73, 74, 75, 76, 77, 78, 79, 82, 84, 87, 88, 89, 92, 94, 95
		Credentials	26 (26.80%)	2, 3, 7, 15, 17, 25, 26, 41, 42, 44, 45, 46, 48, 49, 55, 57, 58, 59, 62, 70, 72, 78, 86, 88, 89, 90
		Occupation	31 (31.96%)	2, 3, 5, 7, 10, 17, 19, 22, 24, 26, 32, 34, 35, 41, 43, 46, 49, 53, 55, 58, 60, 62, 67, 71, 77, 78, 82, 87, 88, 89, 97
		Gender	44 (45.36%)	2, 3, 5, 7, 10, 15, 17, 19, 22, 24, 26, 33, 34, 35, 41, 42, 43, 44, 45, 46, 48, 49, 50, 51, 52, 55, 59, 60, 65, 67, 68, 71, 75, 76, 77, 78, 79, 80, 86, 87, 89, 93, 96, 97
		Experience and training	38 (39.18%)	1, 2, 3, 4, 7, 10, 12, 15, 17, 19, 21, 22, 25, 36, 37, 38, 48, 49, 53, 57, 60, 62, 64, 65, 69, 70, 72, 74, 77, 78, 81, 82, 85, 87, 89, 92, 96, 97
	Relationship with participants	Relationship established	0 (100%)	
		Participant knowledge of the interviewer	3 (3.09%)	75, 78, 89
		Interviewer characteristics	10 (10.31%)	2, 5, 41, 45, 46, 48, 68, 78, 84, 89
**Domain 2: Study Design**	Theoretical framework	Methodological orientation and Theory	64 (65.98%)	1, 2, 4, 5, 8, 9, 10, 12, 15, 17, 22, 23, 25, 30, 31, 32, 34, 36, 38, 40, 41, 42, 43, 44, 45, 46, 47, 48, 51, 52, 53, 54, 55, 57, 59, 60, 61, 62, 63, 64, 65, 67, 69, 70, 72, 73, 74, 75, 76, 77, 78, 79, 80, 82, 83, 85, 87, 88, 90, 91, 92, 93, 94, 97
	Participant Selection	Sampling	92 (94.85%)	1, 2, 3, 4, 5, 6, 7, 8, 9, 10, 11, 12, 13,14,15, 16, 17, 18, 19, 20, 21, 22, 23, 24, 25, 26, 27, 28, 29, 30, 31, 32, 33, 34, 35, 36, 37, 38, 39, 40, 41, 42, 43, 44, 45, 46, 47, 48, 49, 50, 51, 52, 53, 54, 55, 56, 57, 58, 59, 60, 61, 62, 63, 64, 65, 66, 67, 68, 69, 70, 71, 72, 73, 74, 75, 76,

				77,78, 80,81, 83, 85, 86, 88, 89, 91, 92, 93, 94, 95, 96 97
		Method of approach	88 (90.72%)	1, 2, 3, 4, 5, 6, 7, 8, 10, 11, 12, 13,14,15, 16, 17, 18, 19, 21, 23, 24, 25, 26, 27, 28, 29, 30, 31, 32, 34, 36, 37, 38, 39, 40, 41, 42, 43, 44, 45, 46, 47, 48, 49, 50, 51, 52, 53, 54, 55, 56, 57, 58, 59, 60, 61, 62, 63, 64, 65, 66, 67, 68, 69, 70, 71, 72, 73, 74, 75, 76, 77, 78, 79, 81, 82, 86, 87, 88, 89, 90, 91, 92, 93, 94, 95, 96, 97
		Sample size	97 (100%)	1, 2, 3, 4, 5, 6, 7, 8, 9, 10, 11, 12, 13,14,15, 16, 17, 18, 19, 20, 21, 22, 23, 24, 25, 26, 27, 28, 29, 30, 31, 32, 33, 34, 35, 36, 37, 38, 39, 40, 41, 42, 43, 44, 45, 46, 47, 48, 49, 50, 51, 52, 53, 54, 55, 56, 57, 58, 59, 60, 61, 62, 63, 64, 65, 66, 67, 68, 69, 70, 71, 72, 73, 74, 75, 76, 77, 78, 79, 80, 81, 82, 83, 84, 85, 86, 87, 88, 89, 90, 91, 92, 93, 94, 95, 96, 97
		Non-participation	29 (29.90%)	1, 2, 3, 5, 14, 19, 23, 26, 27, 30, 32, 36, 45, 46, 47, 52, 54, 56, 57, 61, 65, 74, 78, 82, 83, 88, 89, 90, 93
	Setting	Setting of data collection	78 (80.41%)	2, 3, 4, 5, 7, 8, 10, 12, 13,14, 17, 18, 20, 21, 22, 23, 24, 25, 26, 27, 28, 30, 32, 33, 34, 35, 38, 39, 40, 41, 43, 44, 45, 46, 47, 48, 50, 52, 53, 54, 55, 57, 58, 59, 60, 61, 62, 63, 65, 67, 68, 69, 70, 71, 72, 73, 74, 75, 76, 77, 78, 79, 80, 81, 82, 83, 84, 86, 87, 88, 89, 90, 91, 92, 93, 94, 96, 97
		Presence of non-participants	16 (16.49%)	5, 12, 15, 16, 18, 21, 25, 35, 44, 54, 63, 66, 71, 74, 77, 96
		Description of sample	93 (95.88%)	1, 2, 3, 4, 5, 6, 7, 8, 9, 10, 11, 12, 13,14,15, 16, 17, 18, 19, 20, 21, 22, 23, 24, 25, 26, 27, 28, 30, 31, 32, 33, 34, 35, 36, 37, 38, 39, 40, 41, 42, 43, 44, 45, 47, 48, 49, 50, 51, 52, 53, 55, 56, 57, 58, 59, 60, 61, 62, 63, 64, 65, 66, 67, 68, 69, 70, 71, 72, 73, 74, 75, 76, 77, 78, 79, 80, 81, 82, 83, 84, 85, 86, 87, 88, 90, 91, 92, 93, 94, 95, 96, 97
	Data Collection	Interview guide	97 (100%)	1, 2, 3, 4, 5, 6, 7, 8, 9, 10, 11, 12, 13,14,15, 16, 17, 18, 19, 20, 21, 22, 23, 24, 25, 26, 27, 28,

				29, 30, 31, 32, 33, 34, 35, 36, 37, 38, 39, 40, 41, 42, 43, 44, 45, 46, 47, 48, 49, 50, 51, 52, 53, 54, 55, 56, 57, 58, 59, 60, 61, 62, 63, 64, 65, 66, 67, 68, 69, 70, 71, 72, 73, 74, 75, 76, 77, 78, 79, 80, 81, 82, 83, 84, 85, 86, 87, 88, 89, 90, 91, 92, 93, 94, 95, 96, 97
		Repeat interviews	1 (1.03%)	55
		Audio/visual recording	94 (96.91%)	1, 2, 3, 4, 5, 6, 7, 8, 9, 10, 11, 12, 13, 14,15, 16, 17, 18, 19, 20, 21, 22, 23, 24, 25, 26, 27, 28, 30, 31, 32, 33, 34, 35, 36, 37, 38, 39, 40, 41, 42, 43, 44, 45, 46, 48, 49, 50, 51, 52, 53, 54, 55, 56, 57, 58, 59, 60, 61, 62, 63, 64, 65, 66, 67, 68, 69, 70, 71, 72, 73, 74, 75, 76, 77, 78, 79, 80, 81, 82, 83, 84, 85, 86, 87, 88, 89, 90, 91, 92, 94, 95, 96, 97
		Field notes	23 (23.71%)	2, 5, 12, 15, 16, 18, 24, 25, 41, 42, 49, 54, 55, 69, 72, 77, 78, 80, 82, 86, 89, 91, 94
		Duration	70 (72.16%)	2, 4, 5, 7, 8, 10, 12, 13, 15, 17, 19, 20, 22, 23, 24, 25, 26, 30, 31, 33, 34, 35, 37, 38, 39, 40, 41, 42, 43, 45, 48, 50, 51, 52, 53, 54, 55, 57, 58, 59, 60, 62, 64, 65, 67, 69, 70, 71, 72, 74, 75, 76, 77, 78, 79, 80, 81, 82, 83, 85, 86, 87, 88, 89, 90, 91, 92, 93, 94, 97
		Data saturation	46 (47.42%)	1, 5, 7, 8, 9, 10, 15, 17, 19, 22, 24, 26, 27, 30, 32, 34, 35, 37, 43, 45, 47, 48, 51, 52, 54, 55, 59, 61, 62, 63, 64, 70, 75, 78, 79, 80, 82, 85, 87, 88, 89, 91, 92, 93, 94, 96
		Transcripts returned	5 (5.15%)	19, 23, 26, 48, 92
**Domain 3: Analysis and Findings**	Data Analysis	Number of data coders	69 (71.13%)	1, 2, 3, 4, 5, 6, 8, 9, 10, 12, 14, 15, 16, 17, 19, 20, 22, 23, 24, 25, 26, 31, 33, 34, 35, 36, 37, 38, 42, 43, 45, 46, 47, 49, 56, 57, 58, 59, 60, 61, 62, 63, 64, 65, 67, 68, 69, 71, 72, 73, 74, 76, 77, 78, 79, 82, 83, 84, 85, 86, 87, 88, 89, 90, 92, 94, 95, 96, 97
		Description of the coding tree	51 (52.58%)	2, 4, 5, 6, 8, 9, 10, 11, 12, 13, 15, 16, 19, 20, 22, 23, 25, 26, 28, 29, 31, 32, 34, 38, 40, 41, 42, 44, 45, 47, 48, 49, 51, 57, 60, 61, 62, 63, 64, 65, 68, 70, 72, 73, 76, 85, 88, 89, 92, 93, 94

		Derivation of themes	90 (92.78%)	1, 2, 3, 4, 5, 6, 7, 8, 9, 10, 11, 12, 13,14,15, 16, 17, 18, 19, 20, 22, 23, 24, 27, 28, 29, 30, 32, 33, 34, 35, 36, 37, 38, 39, 40, 41, 42, 43, 44, 45, 47, 48, 49, 51, 52, 53, 54, 55, 56, 57, 58, 59, 60, 61, 62, 63, 64, 65, 67, 68, 69, 70, 71, 72, 73, 74, 75, 76, 77, 78,79, 80, 81, 82, 83, 84 85, 86, 87, 88, 89, 90, 91, 92, 93, 94, 95, 96, 97
		Software	63 (64.95%)	1, 3, 4, 5, 7, 8, 9, 10, 11, 12, 13, 14, 15, 16, 17, 18, 19, 20, 21, 22, 25, 27, 28, 29, 30, 31, 32, 34, 35, 37, 42, 44, 45, 47, 49, 51, 53, 54, 55, 56, 61, 63, 64, 67, 68, 70, 71, 72, 73, 74, 75, 78, 82, 84, 86, 88, 89, 90, 92, 93, 94, 95, 96
		Participant checking	9 (9.28%)	9, 16, 19, 26, 27, 28, 31, 44, 48
	Reporting	Quotations presented	97 (100%)	1, 2, 3, 4, 5, 6, 7, 8, 9, 10, 11, 12, 13,14,15, 16, 17, 18, 19, 20, 21, 22, 23, 24, 25, 26, 27, 28, 29, 30, 31, 32, 33, 34, 35, 36, 37, 38, 39, 40, 41, 42, 43, 44, 45, 46, 47, 48, 49, 50, 51, 52, 53, 54, 55, 56, 57, 58, 59, 60, 61, 62, 63, 64, 65, 66, 67, 68, 69, 70, 71, 72, 73, 74, 75, 76, 77, 78, 79, 80, 81, 82, 83, 84, 85, 86, 87, 88, 89, 90, 91, 92, 93, 94, 95, 96, 97
		Data and findings consistent	97 (100%)	1, 2, 3, 4, 5, 6, 7, 8, 9, 10, 11, 12, 13,14,15, 16, 17, 18, 19, 20, 21, 22, 23, 24, 25, 26, 27, 28, 29, 30, 31, 32, 33, 34, 35, 36, 37, 38, 39, 40, 41, 42, 43, 44, 45, 46, 47, 48, 49, 50, 51, 52, 53, 54, 55, 56, 57, 58, 59, 60, 61, 62, 63, 64, 65, 66, 67, 68, 69, 70, 71, 72, 73, 74, 75, 76, 77, 78, 79, 80, 81, 82, 83, 84, 85, 86, 87, 88, 89, 90, 91, 92, 93, 94, 95, 96, 97
		Clarity of major themes	96 (98.97%)	1, 2, 3, 4, 5, 6, 7, 8, 9, 10, 11, 12, 13,14,15, 16, 17, 19, 20, 21, 22, 23, 24, 25, 26, 27, 28, 29, 30, 31, 32, 33, 34, 35, 36, 37, 38, 39, 40, 41, 42, 43, 44, 45, 46, 47, 48, 49, 50, 51, 52, 53, 54, 55, 56, 57, 58, 59, 60, 61, 62, 63, 64, 65, 66, 67, 68, 69, 70, 71, 72, 73, 74, 75, 76, 77, 78, 79, 80, 81, 82, 83, 84, 85, 86, 87, 88, 89, 90, 91, 92, 93, 94, 95, 96, 97
		Clarity of minor themes	42 (43.30%)	1, 2, 3, 4, 5, 10, 19, 23, 28, 31, 33, 34, 39, 41, 42, 44, 48, 50, 52, 57, 59, 60, 66, 71, 74, 76,

				78, 79, 80, 81, 82, 83, 83, 84, 85, 86, 87, 88, 89, 90, 91, 92
