# Supplementary material for: Vaccine preferences and their role for vaccine confidence and uptake: a meta-ethnography
Source: Glob Health Action. 2026 Feb 13;19(1):2588846. doi: 10.1080/16549716.2025.2588846 (PMC12912219; doi:10.1080/16549716.2025.2588846)
Supplement: Supplementary Table 2_Characteristics of Included Studies_Clean Copy_26 Aug 2025.docx [file ZGHA_A_2588846_SM7903.docx]

| No. | Author  (Year of Publication) | Qualitative Research  Design | Country | | Research Question | Sample Population | Sampling Methods Employed | Data Collection Method | Analytical Approach |
| --- | --- | --- | --- | --- | --- | --- | --- | --- | --- |
| 1 | Enkel et.al. (2018) | Constructivism | | Australia | To identify the behaviours, knowledge and attitudes of ‘hesitant compliers’ in Perth, Western Australia, | Parents of children under 5 years old (n = 9) | Purposive sampling | Interviews (In-depth interview (IDI)) | Inductive Inquiry Method |
| 2 | Bell et.al. (2019) | Descriptive Qualitative Study | | United Kingdom | To explore vaccination attitudes and behaviours among Polish and Romanian communities, and related access to primary healthcare services. | Polish and Romanian community members (CMs) and Healthcare workers (HCWs) (n=50) | Purposive and Snowball sampling | Interviews (IDI) | Framework Analysis |
| 3 | Sim et.al. (2011) | Phenomenology | | United Kingdom | To assess how pregnant Polish migrants to Scotland weighed up the risks and benefits of the vaccine for pandemic H1N1 influenza in comparison with their Scottish counterparts. | Polish and Scottish women (n=10) | Purposive sampling | Interviews | Thematic Analysis |
| 4 | Ko et.al. (2019) | Narrative | | USA | To determine how Human Papillomavirus (HPV) vaccine perceptions and uptake are shaped among Somali, Ethiopian, and Eritrean mothers. | Somali, Amharic or Tigrinya mothers of 11–17 years old children (n=30) | Purposive sampling | Focus Groups | Framework Analysis |
| 5 | Romijnders et.al (2019) | Descriptive Qualitative Research | | The Netherlands | To explore the factors that play a role in informed decision-making about childhood vaccination | Parents of children aged 2 years old (born in 2013 and 2014) who are classified as acceptor, refusers and partial acceptors (n=55) | Purposive sampling | Focus Groups | Thematic analysis |
| 6 | Remes et.al. (2012) | Formative Qualitative Study | | Tanzania | To better understand potential barriers and facilitators of HPV vaccination and guide immunization programs. | Parents, female pupils, teachers, HCWs and religious leaders (n=169) | Purposive sampling | Interviews and Focus groups | Thematic Analysis |
| 7 | Evans et.al. (2007) | Grounded Theory | | United Kingdom | To investigate lay beliefs about influenza and influenza vaccine in older people to identify appropriate ways of promoting vaccine uptake. | People aged 65 years and over (n=54) | Purposive sampling | Interviews (Narrative interviews) | Analytic induction |
| 8 | Rumetta et.al. (2020) | Descriptive Qualitative Study | | Malaysia | To explore parents’ reasons for refusal of childhood vaccinations in Malaysia and their recommendations on addressing their concerns. | Parents who refused any type of childhood vaccination and resided in Klang Valley (n=14) | Purposive sampling and snowballing | Interviews (IDI) | Framework Analysis |
| 9 | Newman et. al. (2009) | Interpretive/ Cognitive Qualitative research | | USA | To explore HIV vaccine mental models among adults from segments of the population disproportionately affected by HIV/AIDS. | Adults from segments of the population disproportionately affected by HIV/AIDS. (n=99) | Purposive venue-based sampling | Focus Groups | Narrative Thematic Analysis |
| 10 | Fleming et. al. (2020) | Ethno-Epidemiological study | | Canada | To explore the perceptions of People who used drug (PWUD) towards a hypothetical HIV vaccine in Vancouver, Canada, and their implications for the acceptability and uptake of an efficacious vaccine. | PWUD (n=25) | Snowball sampling and street outreach | Interviews (IDI) | Thematic Analysis |
| 11 | Katahoire et. al. (2013) | Descriptive Qualitative Study | | Uganda | To explore the acceptability of HPV vaccine among girls aged 10 to 15 years during 2008 and 2009. | Fully, partially, and non- vaccinated girls aged 10 to 15 years (during 2008 and 2009) (n=422) | Purposive sampling | Focus Groups | Thematic analysis |
| 12 | Reiter et.al (2014) | Descriptive Qualitative Study | | USA | To examine Appalachian communities’ acceptability of HPV vaccine for males and potential barriers to vaccinating males against HPV in their communities. | Health care providers, community leaders, parents with adolescent sons, and young adult women ages 18 to 26 years (n=102) | Purposive Sampling | Focus groups and Interviews (IDI) | Content Analysis |
| 13 | Paul et. al. (2014) | Exploratory Qualitative Study | | India | To examine parental attitudes towards HPV vaccine. | Parents with daughter(s) under 18 years old (n= 36) | Purposive and Convenience Sampling | Interviews | Thematic Analysis |
| 14 | Bair et. al. (2008) | Descriptive  Qualitative Study | | USA | To describe Latina mothers’ acceptance of the HPV vaccine for their daughters and explore their knowledge base regarding HPV-related issues. | Latina mothers of daughters 7 - 14 years old (n = 40) | Convenience Sampling | Interviews | Thematic Analysis |
| 15 | Katz et. al. (2009) | Descriptive Qualitative Study | | USA | To gain insight into the factors that might affect HPV acceptance at the individual and community level in Ohio Appalachia. | Women (18–26 years), parents, community leaders, and healthcare providers (n=114) | Purposive Sampling | Focus groups and Interviews | Framework  Analysis |
| 16 | Cover et. al. (2012) | Descriptive Qualitative Study | | Vietnam | To explore parents’ rationales for vaccinating or not not vaccinating their daughters (vaccine acceptance) and their decision-making process in the context of an HPV vaccination demonstration in project in Vietnam | Parents of girls eligible for vaccination (i.e. Grade 6 or 11 years old) (n=133) | Criteria-based purposive sampling and random sampling | Focus groups. and Interviews | Thematic Analysis |
| 17 | Fadda et. al. (2015) | Phenomenology | | Switzerland | To explore the factors driving parental MMR vaccination decision with regards to vaccination literacy and psychological empowerment. | Parents of at least one child under the age of 12 months (n=20) | Purposive Sampling | Interviews | Thematic Analysis |
| 18 | Singh et. al. (2019) | Multi-level Qualitative Approach | | India | To explore major barriers, potential opportunities, and key facilitators of childhood immunization in slums | NPOs of immunization sessions (n=10),  IDIs of influencers in the family, healthcare service providers, policy makers, and policy influencers (n=65) | Purposive sampling | Observation and Interviews | Thematic Analysis |
| 19 | Siu (2013) | Descriptive Qualitative Study | | Hong Kong Special Administrative Region | To investigate the barriers to receiving HPV vaccine among female undergraduate students in a Hong Kong university. | Young women aged 19 to 23 (n= 35) | Purposive sampling | Interviews (IDI) | Thematic Content Analysis |
| 20 | Burghouts et.al. (2017) | Descriptive Qualitative Study | | Venezuela | To gain insight into reasons for vaccine acceptance or rejection among Warao Amerindians in Venezuela. | Primary caregivers of children aged 6 weeks– 6 months (n=30) | Purposive Sampling | Interviews (IDI) | Thematic Analysis |
| 21 | Flood et.al. (2011) | Descriptive Qualitative Study | | USA | To explore children’s perceptions of influenza, preferences for influenza vaccines, and ability to understand ‘‘risk’’ of vaccine adverse effects and different attributes between injectable and intranasal vaccines.; | Children aged 6 through 12 years (n = 28) | Purposive Sampling | Interviews | Thematic Analysis |
| 22 | Wang et. al. (2014) | Grounded Theory | | Hong Kong Special Administrative Region | To explored how a subsample of new immigrant mothers from mainland China made decisions on various childhood and adolescent vaccines for their offspring, and identified key influences affecting their decision making. | Chinese new immigrant mothers with at least one child aged 14 years or younger living in a Hong Kong household (n = 23) | Purposive Sampling | Interviews (IDI) | Constant Comparative Analysis |
| 23 | Hopfer and Clippard (2011) | Narrative Inquiry | | USA | To increase understanding of the meaning’s college women ascribe to human papillomavirus (HPV) vaccine messages they receive from family, peers, and health care providers | College women (n= 36);  college health clinicians (n = 2) | Purposive Sampling | Interviews (IDI) | Constant Comparative Analysis |
| 24 | Winslade et. al. (2017) | Cross-sectional Qualitative Study | | United Kingdom | To gain a better understanding of London women’s views and experiences on pertussis vaccination and to identify how services might be improved | Mothers (n =42) | Purposive sampling | Interviews | Thematic Analysis |
| 25 | Ramanadhan et. al. (2020) | Descriptive Qualitative Study | | USA | To explore alternative delivery mechanisms, specifically delivery of the vaccine in community settings. | adolescents aged 11–14 who had not received the HPV vaccine yet and caregivers of adolescents (n=41) | Purposive sampling | Focus groups | Framework analysis |
| 26 | Tarrant M. & Gregory D. (2002) | Exploratory Qualitative  Study | | Canada | To explore First Nations parents’ beliefs about childhood immunizations and examined factors influencing immunization uptake. | Mothers of less than 5 years old children from two First Nations (Native Indians) communities (n= 28) | Purposive sampling | Interviews (IDI) | Content analysis |
| 27 | Berman et.al. (2017) | Qualitative Survey Study | | Canada | To survey the belief and attitudes for the two available PCVs in healthcare professionals and immunization experts | Members of Canadian immunization committees and/or participants working in frontline healthcare delivery  (n = 21) | Stratefied Purposive sampling | Interviews | Thematic analysis |
| 28 | Pugliese-Garcia et.al. (2018) | Rapid Qualitative Assessment | | Zambia | To investigate the overarching perceptions on vaccine acceptability, hesitancy, and accessibility at three informal settlements in Lusaka, Zambia. | Laypersons, lay healthcare workers, neighborhood health committee members and vaccinators (n=460) | Convenience sampling | Focus groups | Content analysis |
| 29 | Kanagat et.al. (2020) | Formative Qualitative Study | | Senegal, Vietnam, and Zambia | To explore HCWs’ behavior regarding opening vials and their perceptions and preferences for the number of doses in vials of BCG and measles-containing vaccine (MCV). | Health care workers (n=90) | Stratefird rndom sampling (Senegal);  Purposive sampling (Vietnam); Phased district – level selection (Zambia) | Interviews (KIIs) | Thematic Anaysis |
| 30 | Rubens-Augustson et.al (2019) | Descriptive Qualitative Study | | Canada | To explore the experiences and perceptions of healthcare providers who administer the HPV vaccine to newcomers in Ottawa, Ontario. | Healthcare providers (n=10) | Purposive sampling | Interviews | Content analysis |
| 31 | Newman et.al. (2012) | Grounded Theory | | Canada | To explore HIV vaccine acceptability and strategies for culturally appropriate dissemination among sexually diverse Aboriginal peoples in Canada, among those at highest HIV risk. | Aboriginal male and female, service users, peer educators and service providers (n=23) | Purposive sampling | Focus groups | Narrative Thematic analysis |
| 32 | Collins et.al. (2014) | Descriptive Qualitative Study | | Australia | To gain an in-depth understanding of the decision-making process and factors influencing a pregnant woman’s decisions about recommended immunizations. | Pregnant women (n=17) | Purposive sampling | Interviews | Thematic analysis |
| 33 | Ambali et.al. (2022) | Descriptive Qualitative Study | | Nigeria | To assess mothers’ acceptability and concerns regarding uptake of HPV vaccine by their adolescent daughters. | Mothers of adolescent girls aged 9–15 years (n=20) | Snowball sampling | Interviews (IDI) | Thematic analysis |
| 34 | Tuckerman et.al (2020) | Descriptive Qualitative Study | | Australia | To identify the vaccination decision making, provider practices and perceived barriers and facilitators to recommending or delivering influenza vaccine for children with SRMCs at the tertiary and primary care levels | Medical practitioners (n=26) | Purposive sampling | Interviews | Thematic analysis |
| 35 | McIntyre et.al. (2014) | Exploratory Qualitative study | | Canada | To explore the self-perceived influences among older adults in deciding whether to take or not take the seasonal influenza vaccine. | Receivers and non-receivers of seasonal influenza vaccine aged 67–91(n=31) | Purposive sampling | Focus groups | Content analysis |
| 36 | Kwong et.al. (2010) | Explorartory  Qualitative study | | South Korea, Canada, United Kingdom, Greece, Brazil, Turkey, China, Nigeria and Indonesia | To explore the factors that influence older people’s preferences and uptake of the influenza vaccine in nine countries with a variety of cultures, economic status and vaccination coverage. | Older perople from Chinam Indonesia, Turkey, Korea, Greece, Canada, the United Kingdom, Brazil and Nigeria (n=208) | Purposive sampling | Focus groups | Thematic content analysis |
| 37 | Visser et. al. (2016) | Exploratory Qualitative Study | | The Netherlands | To explore the perceived determinants (barriers and facilitators) of intention to accept vaccination among the possible target groups of pertussis vaccination for cocooning | Parents, maternity, assistants, midwives and paediatric nurses (n=65) | Purposive sampling | Focus groups and Interviews | Thematic content analysis |
| 38 | Wilson et. al. (2013) | Descriptive Qualitative Study | | USA | To examine HPV vaccine knowledge and acceptability among ethnically diverse Black women. | Black women (n=44) | Purposive sampling | Focus groups | Thematic content analysis |
| 39 | McClelland et.al. (2006) | Exploratory Qualitative Study | | Australia | To explore knowledge of and attitudes toward sexually transmissible infections, HPV vaccination and vaccine acceptability among young people in Australia and the factors influencing acceptance and attempts to identify gender differences in knowledge and acceptance. | Young men and women aged between 18 and 23 years (n=14) | Purposive sampling | Interviews (IDI) | Thematic analysis |
| 40 | McComb et. al. (2018) | Exploratory Qualitative Study | | Canada | To explore knowledge, attitudes and barriers regarding the HPV vaccine of immigrant women | Immigrant and refugees women ages 18–26 years (n=11) | Purposive sampling | Interviews | Thematic content analysis |
| 41 | Waller et.al. (2006) | Exploratory Qualitative Study | | United Kingdom | To explore mothers’ attitudes towards HPV vaccine. | Mothers of daughters ages 8 to 14 years (n=24) | Purposive sampling | Focus groups | Framework analysis |
| 42 | Eilers et.al. (2015) | Descriptive Qualitative Study | | The Netherlands | To elucidate the motives of Dutch persons aged ≥50 years for accepting vaccination. | Individuals 50 years or older (n=80) | Convenience/ Purposive sampling | Focus groups | Thematic analysis |
| 43 | Bland et.al. (2009) | Descriptive Qualitative Study | | New Zealand | To identify the factors that influenced young adults (aged 16–19 years) when deciding whether or not to be immunised during the MeNZB campaign. | Young adults aged 16-19 years (n=11) | Purposive sampling | Interviews | Thematic analysis |
| 44 | Jackson et.al. (2017) | Cross-sectional Qualitative Study | | United Kingdom | To investigate the views of Travellers in the UK on the barriers and facilitators to acceptability and uptake of immunisations and explore their ideas for improving immunisation uptake and to examine whether and how these responses vary across and within communities, and for different vaccines (childhood and adult) | Travellers: (Romanian Roma, English Gypsy/Irish Travellers (Bristol), English Gypsy (York), Romanian/Slovakian Roma, Scottish Show people (Glasgow) and Irish Traveller (London) (n=174) | Purposive sampling and snowball sampling | Focus groups and Interviews (IDI) | Framework anlysis |
| 45 | Westrick et.al. (2017) | Descriptive Qualitative Study | | USA | To determine parents’ knowledge and attitudes regarding human papillomavirus (HPV) vaccinations in their adolescent children and to describe parents’ perceptions of adolescent vaccinations in community pharmacies | Parents or guardians of children ages 11–17 years (n=26) | Purposive sampling | Interviews (IDI) | Thematic analysis |
| 46 | Sampson et. Al. (2011) | Exploratory Qualitative Study | | United Kingdom | This study aimed to develop a better understanding of the reasons for poor uptake (in those aged 2–16 years in an at-risk group), in order to inform ways of improving rates of vaccination. | Parents of children age 2-16 years (n=38) | Purposive sampling | Qualitative survey with follow-up interviews | Thematic analysis |
| 47 | Krawczyk et. Al. (2015) | Qualitative Survey Study | | Canada | To examine the reasons given by parents who accepted or refused the HPV vaccine for their daughters in the context of a free provincial school-based vaccination program | Parents of 9– 10 y old girls (n=806) | Random sampling | Qualitative survey | Framework analysis |
| 48 | Gottvall et.al. (2017) | Exploratory Qualitative Study | | Sweden | To explore parents’ views of extending the human papillomavirus (HPV) vaccination programme to also include boys | Parents who were offered HPV vaccination for their 11–12 years old daughter in the national school-based vaccination programme (n = 42) | Purposive sampling | Interviews | Thematic content analysis |
| 49 | Jalloh, et. al. (2019) | Formative Qualitative  Study | | Sierra Leone | To examined hypothetical acceptability and perceptions of experimental Ebola vacci nes among HCWs, frontline workers, and the general public to guide ethical communication of risks and benefits of any experimental Ebola vaccine. | Public Health Leaders, HCWs, Frontline Workers and General Public (n=66) | Purposive sampling | Focus groups and Interviews | Content analysis |
| 50 | Siu (2014) | Exploratory Qualitative Study | | Hong Kong Special Administrative Region | To investigate the perceptions of Hong Kong mothers in regard to vaccinating their daughters against HPV in Hong Kong | Mothers aged 30 to 60 years old with daughter(s) between 9 and 17 years old (n = 35) | Purposive sampling | Interviews | Thematic analysis |
| 51 | Charania et.al. (2018) | Exploratory Qualitative Study | | New Zealand | To understand the perceptions of caregivers and health-care providers regarding the potential introduction of routine varicella vaccination. | Caregivers and health-care providers (n = 20) | Purposive sampling | Interviews | Thematic Analysis |
| 52 | Yuen et.al. (2016) | Descriptive  Qualitative Study | | Hong Kong Special Administrative Region | To explore Hong Kong women’s perceptions of the threat of influenza infection during pregnancy, the risks and benefits of influenza vaccination, and their decision-making processes. | Women who had just given births to a live infant from April to June 2011 (n=32) | Purposive sampling | Interviews | Thematic analysis |
| 53 | Kobetz, et.al. (2011) | Exploratory Qualitative Study | | Haiti | To examine Haitian women’s perceptions of, and barriers to, HPV vaccination. | Haitian women currently living in little Haiti and aged 21 to 75 (n=41) | Communitybased recruitment | Focus Groups | Framework analysis |
| 54 | Turiho, et.al (2017) | Exploratory  Qualitative Study | | Uganda | To explore community member’s perceptions about HPV vaccination in Ibanda district and the implications of the perceptions for acceptability of HPV vaccination | School girls, Parents, Health Workers, Community Leaders and Teachers (n=105) | Purposive sampling | Focus groups and Interviews (KII) | Thematic analysis |
| 55 | Benin et.al. (2006) | Descriptive Qualitative Study | | USA | To investigate decision-making about vaccinations for infants | Mothers 1 to 3 days postpartum and again at 3 to 6 months (n=33) | Purposive sampling | Interviews | Thematic analysis |
| 56 | Paterson et.al. (2018) | Cross-sectional Qualitative Study | | United Kingdom | To explore the reasons some parents decided not to vaccinate their child against influenza as part of the pilot programme offered in schools | Parents whose children were not vaccinated against influenza (n = 25) | Purposive sampling | Interviews | Thematic analysis |
| 57 | Meharry et.al. (2013) | Descriptive Qualitative Study | | USA | To gain an in-depth understanding of the reasons why pregnant women accept or reject the seasonal influenza vaccine. | Pregnant women or new mothers (n=60) | Purposive sampling | Interviews | Content Analysis |
| 58 | Padmawiti et.al. (2019) | Exploratory Qualitative Study | | Indonesia | To explore the views of religious and community leaders regarding the rotavirus vaccine to inform future communication strategies. | Religious leaders and Community representatives (n=20) | Purposive sampling | Interviews (IDI) | Thematic analysis |
| 59 | Fernandez-Pineda, et. al. (2020) | Descriptive Qualitative Sudy | | USA | To determine the salient factors among Hispanic parents for vaccinating their children against HPV and for designing a future HPV prevention intervention for Hispanics. | Hispanic parents from rural communities in South Florida (n= 23) | Purposive sampling | Focus Groups | Content analysis |
| 60 | Wheelock, et.al. (2014) | Exploratory Qualitative Study | | United Kingdom | To explore the socio-psychological factors that drive adult vaccination in the UK, specifically influenza and tetanus, and to evaluate whether these factors are comparable between vaccines. | Adult participants (n = 20) | Purposive sampling | Interviews (IDI) | Thematic analysis |
| 61 | Sun et.al. (2018) | Exploratory Qualitative Study | | China | To explore how the stated importance of different disease and vaccine-related attributes interacted with beliefs about the immune system of a child to affect Chinese parents’ decision to obtain a non-EPI vaccine. | Parents of young children at immunization clinics (n=34) | Purposive sampling | Interviews | Thematic analysis |
| 62 | Ganczak, et.al. (2021) | Descriptive Qualitative Study | | Poland | To identify vaccination practices in Ukrainian migrant group, to explore facilitators and barriers to vaccination and related access to Polish healthcare services | Ukrainian migrants (UMs) living in Szczecin, Poland (n=22) | Snowball sampling | Focus Groups | Thematic analysis |
| 63 | Harmsen, et.al. (2015) | Descriptive Qualitative Study | | The Netherlands | To explore factors that influence decision-making among parents with different ethnic backgrounds in the Netherlands. | Mothers of Moroccan, Turkish and other ethnic backgrounds with at least one child aged 0–4 years (n= 33) | Purposive sampling | Focus Groups | Thematic analysis |
| 64 | Kajungu, et.al. (2020) | Descriptive Qualitative Study | | Uganda | To explore maternal knowledge, attitudes, willingness, and beliefs towards maternal immunization among pregnant women in rural Uganda | Pregnant women and Health worker (n=90) | Purposive sampling | Focus Groups and Interviews (KII) | Thematic analysis |
| 65 | Telford and Rogers (2003) | Descriptive Qualitative Study | | United Kingdom | To explore the influences on decision making by elderly people for influenza vaccine uptake. | Patients age 75 years and over (n=20) | Purposive sampling | Interviews (IDI) | Thematic analysis |
| 66 | Kristensen, et.al. (2016) | Descriptive Qualitative Study | | Brazil, China, India, Peru, Philippines, and Tanzania | To capture the opinions of stakeholders working in immunization programs in low and middle-income countries to understand how vaccine products could be improved to better meet their needs and to obtain feedback on specific vaccine product attributes including the number of doses per container and ease of preparing a dose for administration | Immunization stakeholders (n=158) | Purposive sampling | Interviews | Thematic analysis |
| 67 | Biezen, et.al. (2018) | Cross-sectional Qualittive Study | | Australia | To explore the views, attitudes and practices of parents and primary care providers (PCPs) on their knowledge and acceptance of influenza vaccination in children under 5. | Primary care providers and parents (n=80) | Purposive sampling | Focus Groups and Interviews | Thematic analysis |
| 68 | Simas, et.al. (2021) | Exploratory  Qualitative Study | | Mexico | To explore trust, views, and attitudes towards maternal immunization among pregnant women in Mexico explored participants’ experiences with maternal vaccination, as well as how they navigated the health system, searched for information, and made decisions around maternal immunization. | Women from Mexico City and Toluca (n=54) | Purposive sampling | Focus Groups and Interviews | Thematic analysis |
| 69 | Xiong, et.al. (2021) | Exploratory  Qualitative Study | | USA | To identify the knowledge, perceptions, and decision-making processes about HPV vaccinations in the Hmong population, an Asian-American group with increased risks of HPV-related cancers. | Hmong adolescents and parents (n=25) | Purposive sampling | Focus Groups | Thematic analysis |
| 70 | Giduthuri, et.al. (2021) | Rapid Ethnographic Qualitative Survey Study | | India | To assess the fundamental aspects of antenatal influenza vaccination (AIV) acceptance and demand among key stakeholders in urban Pune, India. | Private clinicians and members of their community (n=106) | Purposive sampling | Interviews and Qualitative Survey | Thematic analysis |
| 71 | Colmegna, et.al. (2021) | Descriptive Qualitative Study | | Canada | To assess perspectives of RA patients and healthcare professionals (HCPs) involved in RA care of barriers and facilitators regarding influenza and pneumococcal vaccines. | Patients living with rheumatoid arthritis (RA) and Healthcare Professionals (n=54) | Purposive sampling | Focus Groups and Interviews | Framework analysis |
| 72 | Morales-Campos, et.al. (2021) | Exploratory Qualitative Study | | USA and Mexico | To examine gendered perspectives in knowledge, beliefs, and attitudes about HPV and HPV vaccination from Hispanic parents (mothers and fathers), women of vaccine eligible age (18–26 years old), and women eligible for Pap Test screening (>26 years old) living in two counties along the Texas-Mexico border. | Parents of 11 – 17 year old and young women aged 18 – 26 (n=71) | Purposive sampling | Focus Groups | Thematic content analysis |
| 73 | Mitchell, et.al. (2021) | Descriptive Qualitative Study | | Tanzania | To investigate acceptability of dose-reduction among girls, and parents/guardians of girls, randomised to receive one, two or three doses in an HPV vaccine dose-reduction and immunobridging study (DoRIS trial) in Tanzania. | Daughters aged 9 to 12 y/o and/or parents/guardian (n=37) | Random sampling | Interviews | Framework analysis |
| 74 | Glenn, et.al. (2021) | Exploratory Qualitative Study | | USA | To explore perspectives on HPV vaccination among young adults receiving care at the student health center of a large public university. | Female and male undergraduate and graduate students and health care providers (n= 51) | Purposive sampling | Focus Groups and Interviews | Content analysis |
| 75 | Deal, et.al. (2021) | Exploratory Qualitative Study | | United Kingdom | To explore views on the COVID-19 vaccine, including barriers to access, seeking their input into defining action points and developing solutions to strengthen delivery and up take in marginalised migrant communities | Migrants (n=32) | Purposive and snowball sampling | Interviews (IDI) | Framework analysis |
| 76 | Lockyer, et.al. (2020) | Descriptive Qualitative Study | | United Kingdom | To understand people's COVID-19 beliefs, their interactions with (mis)in formation during COVID-19 and attitudes towards a COVID-19 vaccine. | People from different ethnic groups and areas of Bradford (n=20) | Snowball sampling | Interviews | Reflexive  Thematic analysis |
| 77 | Madhivanan, et.al. (2009) | Descriptive Qualitative  Study | | India | To investigates attitudes toward HPV vaccination among parents of adolescent girls in Mysore, India | Parents of adolescent girls (n=44) | Purposive sampling | Focus Groups | Thematic analysis |
| 78 | Dubé, et al. (2022) | Descriptive Qualitative Study | | Canada | To better understand the determinants of health care providers' vaccination decisions, their views on barriers to COVID-19 vaccine acceptance and proposed solutions, their opinions on vaccine policies, as well as their perceived role in discussing COVID-19 vaccination with patients | Healthcare Providers (n=14) | Purposive sampling | Interviews | Thematic analysis |
| 79 | Steffens, et.al. (2022) | Descriptive Qualitative Study | | Australia | To investigate the barriers and facilitators of acceptance of COVID-19 vaccines in adults with underlying health conditions. | Adults with underlying health conditions (n=15) | Purposive sampling | Interviews | Thematic analysis |
| 80 | Marín-Cos, et al. (2022) | Phenomenology and Grounded Theory | | Spain | To understand pregnant women’s and healthcare workers’ (HCWs) perceptions about maternal vaccines, the primary motivational factors for decision-making regarding the uptake of maternal vaccines, and the participation in clinical trials during pregnancy. | Pregnant women and Health Professionals (n=35) | Convenience sampling | Interviews | Thematic analysis |
| 81 | Tibbels, et al. (2022) | Descriptive Qualitative Study | | Côte d’Ivoire | To identify perspectives on COVID-19 vaccines from those who had direct exposure to COVID-19 as well as those who did not to inform programmatic efforts promoting vaccine uptake in Côte d’Ivoire | Individuals who had recovered from COVID-19, lost a family member, members of the general population and health providers (n= 156) | Purposive sampling | Focus Groups and Interviews | Thematic analysis |
| 82 | Sides, et al. (2020) | Descriptive Qualitative Study | | United Kingdom | To explore attitudes and intentions towards COVID-19 vaccinaton and sources of COVID-19 information across diverse ethic groups in the UK | Individuals from diverse ethnic background (n=100) | Purposive sampling | Focus Groups | Thematic analysis |
| 83 | Shah, P., et al. (2021) | Exploratory Qualitative Study | | India | To assess the awareness, attitudes, and beliefs regarding HPV and HPV vaccination and explore the barriers and challenges to HPV vaccine intent among women in Mangalore, India. | Women aged 18 to 45 y/o (n=22) | Purposive sampling | Focus Groups and Interviews | Thematic analysis |
| 84 | Long, et al (2022) | Descriptive Qualitative Study | | China | To elucidate the factors hindering vaccine uptake, address their modifications since the previous year, and provide policymakers with reference in facilitating booster vaccination to contain the COVID-19 pandemic | Chinese residents (n=60) | Purposive sampling | Interviews | Purposive sampling |
| 85 | Jha, et al. (2022) | Descriptive Qualitative study | | India | To find the perceptions regarding  the vaccine and understand the influencers and the barriers of vaccine acceptance. | Healthcare workers (HCW) and community members (n=18) | Convenience sampling | Interviews | Thematic analysis |
| 86 | Knight., et al. (2021) | Phenomenology | | USA | To understand the facilitators and barriers to COVID-19 testing and vaccine acceptability among homeless-experienced adults | Homeless-experienced older adults (n=94) | Purposive and convenience sampling | Interviews | Content analysis |
| 87 | Bullivant, et al (2023) | Descriptive Qualitative Study | | Australia | To explore older adults’ intention towards COVID-19 vaccination and factors that influenced their motivation to get vaccinated. | Older adults (n=14) | Purposive sampling | Interviews | Thematic analysis |
| 88 | Balasuriya, et al (2021) | Descriptive Qualitative Study | | USA | To investigate and understand factors associated with facilitating and obstructing COVID-19 vaccine access and acceptance among Black and Latinx communities. | 72 participants | Purposive sampling | Focus Groups | Content analysis |
| 89 | Huang,W., et al (2023) | Descriptive Qualitative Study | | USA | To better understand and contextualize COVID-19 vaccine hesitancy among persons from under-represented racial/ethnic populations in the Southern US. | Individuals from under-represented racial/ ethnic populations (n=29) | Purposive sampling | Interviews | Thematic analysis |
| 90 | Carson, et al (2021) | Descrptive Qualitative Study | | USA | To examine factors that members of multiethnic communities at high risk for COVID-19 infection and morbidity report as contributing to vaccine decision-making. | Members of racial and ethnic minority communities (n=70) | Purposive sampling | Focus Groups | Reflexive  Thematic analysis |
| 91 | Duong,M.C., et al (2022) | Descriptive Qualitative Study | | Vietnam | To examine people’s attitudes towards COVID-19 vaccine and associated determinants in Vietnam using qualitative research study, to bring in initiatives to improve the vaccination campaign. | 20 participants | Purposive sampling | Focus Groups | Reflexive  Thematic analysis |
| 92 | Elbarazi,I., et al (2022) | Descriptive Qualitative Study | | Egypt, Qatar, Kingdom of Saudi Arabia, Libya, Sudan, UAE, and Jordan | To explore the enablers and barriers toward vaccine acceptance among Arabs. It investigates the effect of factors like confidence in the healthcare system, misinformation, and scientific approaches adopted to mitigate COVID-19 vaccination among Arabs in different countries. | 100 participants | Convenience and snowball sampling | Focus Groups | Thematic analysis |
| 93 | Ecker,F., et al (2021) | Exploratory Qualitative Study | | Austria | To investigate beliefs and characteristics of vaccine hesitant doctors concerning (i) vac-cination in generally, (ii) specifically the measles vaccine and (iii) their perceptions on measles. | Doctors (n=12) | Conveninece, Purposive and Snowball sampling | Interviews | Content analysis |
| 94 | Yoon,S., et al (2022) | Exploratory Qualitative Study | | Singapore | To explore multifactorial influences that might affect a decision on COVID-19 vaccination and suggestions for decision support to improve vaccine uptake among HCWs in the early phase of vaccination rollout. | Health Care Workers (n=13) | Purposive sampling | Interviews | Thematic analysis |
| 95 | Karafillakis,E., et al (2021) | Exploratory  Qualitative Study | | France, Germany, Italy, Spain, and UK | To explore pregnant women’s experiences, decision-making processes and perceptions towards maternal vaccination and maternal vaccine trials in Europe. | Pregnant women (n=258) | Purposive and Snowball sampling | Focus groups and Interviews | Thematic analysis |
| 96 | Simas, C., et al. (2021) | Exploratory Qualitative Study | | Panama | To investigate views and attitudes towards maternal immunization among pregnant women in Panama City and San Miguelito | Pregnant women (n=56) | Purposive sampling | Focus groups and Interviews (IDI) | Thematic analysis |
| 97 | Fadda, M., et al. (2021) | Exploratory Qualitative Study | | Switzerland | To explore older adults’ attitudes towards and beliefs regarding a future covid-19 vaccination in the Italian-speaking Canton of Ticino in Southern Switzerland. | Older Participant (n=19) | Convenience and Snowball sampling | Interviews | Thematic analysis |

**Supplementary Table 2.** Characteristics of Included Studies
