# Supplementary material for: Vaccine preferences and their role for vaccine confidence and uptake: a meta-ethnography
Source: Glob Health Action. 2026 Feb 13;19(1):2588846. doi: 10.1080/16549716.2025.2588846 (PMC12912219; doi:10.1080/16549716.2025.2588846)
Supplement: Supplementary Table 1_Summary of Research Databases.pdf [file ZGHA_A_2588846_SM7902.pdf]

Supplementary Table 1. Summary of Research Databases

Database		Search String
**1.**	PubMed	(((vaccine preferences) OR (vaccine attributes)) OR (vaccine acceptability)) AND (qualitative research)
**2.**	Science Direct	("vaccine preferences" OR "vaccine attributes" OR "vaccine acceptability") AND ("qualitative research" OR "qualitative study")
**3.**	ProQuest	"Vaccine preferences" OR "vaccine attributes" OR "vaccine acceptability" AND (qualitative study)
**4.**	Ebscohost	"Vaccine preferences" OR "vaccine attributes" OR "vaccine acceptability" AND qualitative research
**5.**	JSTOR	((((("vaccine preferences") OR ("vaccine attributes")) OR ("vaccine acceptability")) AND ("qualitative research")) OR ("qualitative study"))
**6.**	Scopus	"Vaccine preferences" OR "vaccine attributes" OR "vaccine acceptability" AND "qualitative research" OR "qualitative study"
**7.**	Google Scholar	"Vaccine preferences" OR "vaccine attributes" or "vaccine acceptability" AND "qualitative study" OR "qualitative research"
